# A Resonance Approach to Cochlear Mechanics

**DOI:** 10.1371/journal.pone.0047918

**Published:** 2012-11-08

**Authors:** Andrew Bell

**Affiliations:** Eccles Institute of Neuroscience, John Curtin School of Medical Research, The Australian National University, Canberra, Australia; Mount Sinai School of Medicine, United States of America

## Abstract

**Background:**

How does the cochlea analyse sound into its component frequencies? In the 1850s Helmholtz thought it occurred by resonance, whereas a century later Békésy's work indicated a travelling wave. The latter answer seemed to settle the question, but with the discovery in 1978 that the cochlea emits sound, the mechanics of the cochlea was back on the drawing board. Recent studies have raised questions about whether the travelling wave, as currently understood, is adequate to explain observations.

**Approach:**

Applying basic resonance principles, this paper revisits the question. A graded bank of harmonic oscillators with cochlear-like frequencies and quality factors is simultaneously excited, and it is found that resonance gives rise to similar frequency responses, group delays, and travelling wave velocities as observed by experiment. The overall effect of the group delay gradient is to produce a decelerating wave of peak displacement moving from base to apex at characteristic travelling wave speeds. The extensive literature on chains of coupled oscillators is considered, and the occurrence of travelling waves, pseudowaves, phase plateaus, and forced resonance in such systems is noted.

**Conclusion and significance:**

This alternative approach to cochlear mechanics shows that a travelling wave can simply arise as an apparently moving amplitude peak which passes along a bank of resonators without carrying energy. This highlights the possible role of the fast pressure wave and indicates how phase delays and group delays of a set of driven harmonic oscillators can generate an apparent travelling wave. It is possible to view the cochlea as a chain of globally forced coupled oscillators, and this model incorporates fundamental aspects of both the resonance and travelling wave theories.

## Introduction

Over the past 200 years the dominant paradigms in cochlear mechanics have been first the resonance theory, as elaborated by Helmholtz [1] and later the travelling wave theory, developed by Békésy [2]. Both have particular merits, but in the middle of last century the latter prevailed because of Békésy's clear observations of travelling waves in human temporal bones and the seeming impossibility of sustaining the resonance of microscopic tuned elements immersed in fluid. Later theoreticians found that, given appropriate figures for elasticity and mass of the basilar membrane, cochlear mechanics could be fairly well described by a travelling wave of hydrodynamically coupled motion due to pressure differences across it, an effect that propagates along the sensing surface like a ripple on a pond [2]–[4]. This idea of serial excitation in the cochlea has conceptual backing derived from transmission line theory, and observations confirm a wave of activity progressing from base to apex, typically at some metres per second.

But the discovery of otoacoustic emissions by Kemp [5], [6] has changed our understanding of the cochlea immensely. The cochlea is now seen as an active device, and a live cochlea behaves very differently to a dead one. These new findings support the work of Gold [7] who in 1948 conceived of the cochlea as a regenerative receiver, an electronic device that uses positive feedback to overcome damping and increase tuning sharpness. Gold's work opened up an avenue for overcoming the primary obstacle to the Helmholtz resonance theory, a direction that he actively pursued. “[O]nly the resonance theory of Helmholtz” he said, “interpreted in accordance with the considerations [here,] is consistent with observation” [8], p. 462.

However, with no suitable candidates for the resonant elements apart from the basilar membrane itself, the general approach has been to build active properties on top of the passive travelling wave model [6], [9]–[12], a technique that has been more or less successful. There have been many experiments and discussions focusing on the role that this travelling wave plays in cochlear mechanics [13]–[17].

Nevertheless, a number of anomalies remain, and these have been reviewed in ref. [18]. A troublesome feature has been the failure to observe an unmistakable reverse travelling wave, an entity required by current theory in order to recirculate acoustic energy between the peak of the travelling wave and the stapes [6], [19]. According to the theory of coherent reflection filtering [20], [21], this feedback process is required in order to improve tuning and to generate otoacoustic emissions in the ear canal [19]. Some more recent analyses attempt to explain this anomaly in terms of the backward-travelling wave being masked by the simultaneously present forward wave [22], but the special conditions required for this to occur mean that the issue still appears problematic. A related difficulty revolves around the possible role of a fast pressure wave in cochlear mechanics (e.g. refs [23]–[25]). The question is whether energy could travel to and from the hair cells via such a wave, which propagates at the speed of sound in water (1500 metres per second, or nearly instantaneously) through the cochlear fluids. The issue remains open. This paper addresses the issue by considering what the result might be if all the outer hair cells, here represented as a graded bank of uncoupled resonators, were simultaneously excited. The excitation might come directly from the fast pressure wave squeezing the cell body (considered most likely), or by a trans-membrane pressure difference instantaneously deflecting stereocilia (less likely, but possible).

In this way, the paper short-circuits the difficulties inherent in trying to build an active process on top of a passive travelling wave. Instead, the approach is to take the active (resonance-based) process as the prime mechanism and dispense altogether with the travelling wave as a causal agent. This logic inverts the normal causal chain and makes the travelling wave simply a secondary event – an epiphenomenon – that forms in response to the primary active process. Such a travelling wave thus carries no energy as it propagates from base to apex; it is not an effective stimulus, merely the envelope of activity generated by the active resonating elements in direct response to the incoming sound pressure. The active elements, resonantly excited by sound, are precisely those called for by Helmholtz's original resonance theory and later supported by Gold.

Putting the possibility on firmer ground, a candidate for the resonant elements has already been identified [26], and the merits of the resonance approach have been systematically evaluated [18]. Can these non-standard ideas be sustained, and how far is it possible to go by dispensing with a causal travelling wave?

In this paper the approach is to start, for simplicity, with the fundamental resonant unit, the harmonic oscillator, and examine some of its key properties. Certain of these properties – the amplitude and phase response at a point on the basilar membrane, and apparent wave velocity along the membrane – are currently taken as evidence favouring a travelling wave interpretation. This paper will show how these phenomena can largely arise from purely resonant behaviour. A model of the cochlea is assembled in which independent resonant elements form a graded bank of harmonic oscillators driven at their resonant frequencies, much like the piano strings that Helmholtz envisaged, with experimentally determined frequencies and tuning sharpness. They are all simultaneously excited and the response of the system – its amplitude, phase, group delay, and apparent propagation velocity – is examined using basic resonance principles.

The first property examined is the amplitude response of a single resonator and this is shown to be just that of the driven harmonic oscillator, which takes *Q*/*π* cycles to reach a peak. The amplitude profile will be shown to resemble that found in the cochlea. More importantly, the group delay of such a harmonic oscillator is calculated and is shown to amount to several cycles at resonance, an amount typical of the cochlea. This value is far greater than the 180° phase delay typically associated with resonant systems, and normally this 180° figure is taken as conclusive evidence against the validity of resonance theories of hearing (p. 16 of [12]; p. 199 of [11]). Strictly, this is true for pure resonance, but it ignores the possibilities offered by a bank of forced and lightly coupled resonators whose group delays can reach several cycles, and these options are explored further.

The question of cochlear phase delays is addressed here in two ways, firstly by looking at some indicative measures in a mechanical analog (the vibrating reed frequency meter) and secondly by reviewing the literature on chains of phase-coupled oscillators. The literature demonstrates that several cycles of delay are possible, that phase plateaus occur, and that the individual tuned elements in the chain retain an ability to respond resonantly to external forcing.

Another aspect examined is the propagation velocity, a property often taken as the signature of a travelling wave. On the standard view, this sound-induced wave motion is seen as a primary bending stimulus that is progressively delivered to the thousands of hair cells and their projecting stereocilia. However, on the resonance model, when each resonator takes *Q*/π cycles to reach a peak, it produces an envelope of peak displacement which appears to move along the cochlear partition. When the velocity of such a wave is calculated, the values are in line with the travelling wave velocities observed experimentally and with those normally derived from more complex transmission line models.

It is shown that a bank of coupled resonators can readily give the appearance of a travelling wave, but the core of the matter appears to be whether the stimulus energy reaches the detecting elements in series or in parallel. This distinction is vital for deciding whether the cochlea is an essentially resonant system or one driven by travelling waves, and these alternatives are closely examined.

It is concluded that these results, based on fundamental resonator properties and without involving the mass or compliance of the basilar membrane, are more than just coincidence, but rather reflect the basic resonant operation of the cochlea's sensing elements. In other words, the travelling wave velocities that emerge are consistent with a fast pressure wave being the prime stimulus. Travelling wave velocities, then, could simply arise as a secondary manifestation of a fast-acting compression wave acting on tuned elements of specific quality factor, not from a complex interplay of membranes and hydrodynamics.

These outcomes give new life to Helmholtz's theory. They show that much care is needed in deciding whether an observed travelling wave is the result of (i) a stimulus propagating serially along a coupled basilar membrane, or (ii) simultaneous excitation of a graded bank of independent resonators, such as might be produced by a fast pressure wave. The latter situation involves stimulation of all the sensing cells in parallel, not serially. These issues are addressed in the discussion section of this paper and are placed in the framework of the existing literature.

## Analysis: The Driven Harmonic Oscillator

The harmonic oscillator is a fundamental element in both acoustics and electronics and the equations governing it can be found in many textbooks, where it is shown that it can be modeled as either a mass on a spring or an LCR circuit. Here the definitions and derivation found in Fletcher [27] are followed. The electronic approach is taken by Shera [28].

As a starting point, the cochlea is taken to be a graded bank of uncoupled, linear, harmonic oscillators arranged along the basilar membrane and varying in frequency from 20,000 Hz at the base to 20 Hz at the apex, a distance of some 35 mm in humans. This system can be completely specified in terms of the resonant frequency and *Q* of each resonator, and in the case of the cochlea such measures are available.

### A. Quality factor, Q

The harmonic oscillator has a damping coefficient *α* which causes the amplitude of a passive oscillator to decrease. The damping coefficient governs the sharpness of tuning, or quality factor *Q*, of the oscillation. The quality factor is the ratio of the natural frequency of the oscillator, *ω*_0_, divided by the full-width, Δ*ω*, of the response curve at 1/√2 of its height – the half-power criterion [27]. That is,
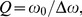
(1)and it follows that *α* = *ω*_0_/2*Q*.

### B. Cochlear Q

The effective *Q* of all the individual resonators is still the subject of on-going research [10], [13], [14]. Different approaches can be taken, but the two main methods are psychophysical (subjective) ones, in which the effective *Q* of the cochlear elements is inferred from masking experiments, and otoacoustic (objective) techniques where the *Q* is derived from studies of the evoked otoacoustic responses of the cochlea to sound impulses. Here we take the results of Shera and colleagues [29] who combined both methods and found empirically that the *Q* of the cochlea at any frequency *f* (in Hz) is given by
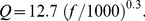
(2)


**Figure 1:**
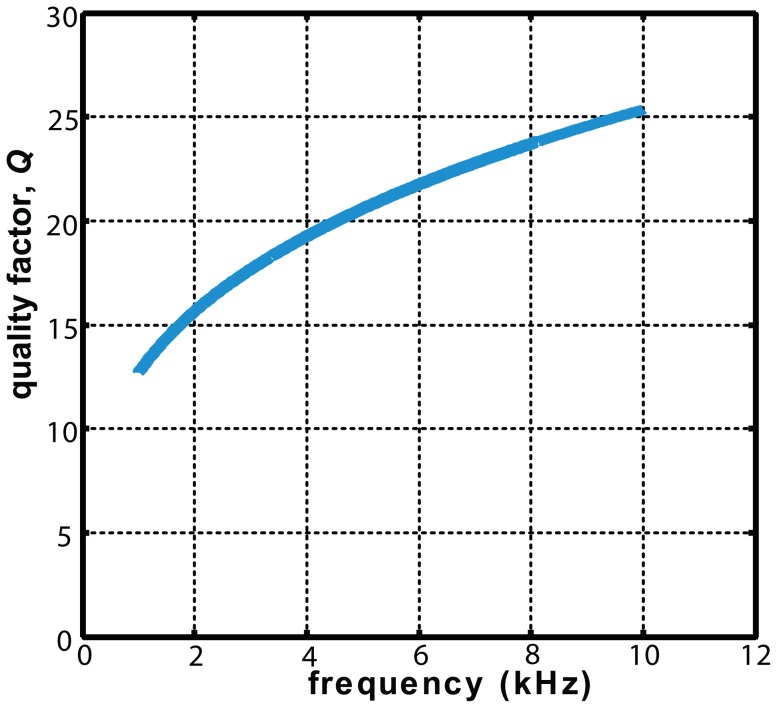
Variation of the cochlea's quality factor with frequency. The line marks the empirical relation between quality factor, *Q*, and frequency, *f*, in kilohertz: *Q* = 12.7 *f* ^0.3^ as determined by Shera *et al.* (2002) [29] from a combination of psychophysical and otoacoustic measurements.

These *Q* values provide a reference point for the present thought experiment. Some have questioned such high values [30], although the criticism has been deemed invalid in a recent review [31]. However, the numerical values used in Eq. (2) are taken as reasonable, and this relation is plotted over the range 1 to 10 kHz in Fig. 1.

### C. Amplitude response of the driven oscillator

**Figure 2:**
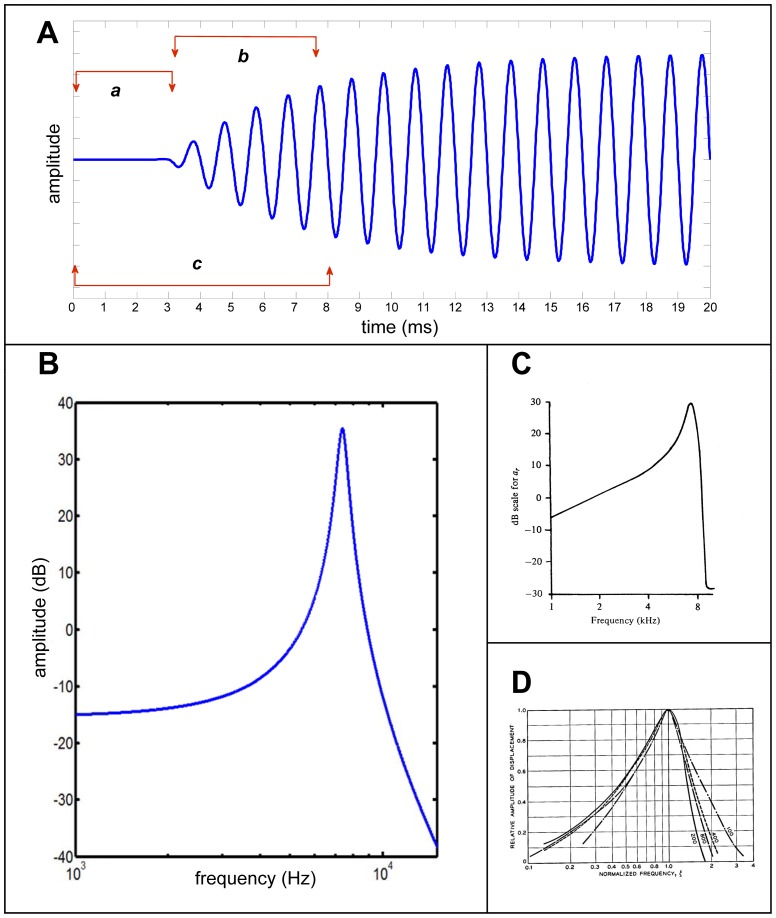
Amplitude response with time and frequency of the driven harmonic oscillator. (**A**) Amplitude response with time when the natural frequency is 1 kHz and the *Q* is 12.7, values reflecting those of the cochlea according to [29]. From the instant of stimulation (at *t* = 3 ms), the oscillator takes *Q*/π cycles (∼4 cycles) to reach the half-power amplitude of 0.707 and *Q* cycles to reach the 0.96 criterion. The labelled bars indicate three delays: *a*, signal-front or propagation delay; *b*, group delay or filter delay (also called resonance build-up time); and *c*, the total delay – their relevance to cochlear mechanics is discussed in the text. (**B**) Amplitude–frequency response (logarithmic axes) of an oscillator with *Q* as before and natural frequency of 7 kHz. (**C**) Typical amplitude response of the cochlea with a CF of 7 kHz, as shown in Fig. 9 of Lighthill (1981) [33]. Note how the response resembles that of the driven oscillator in (B). (**D**) Response of the basilar membrane as recorded by Békésy as travelling waves in the time domain and transformed into the frequency domain by Flanagan (1960) [135]. Last two panels reproduced with permission of Cambridge University Press and Alcatel-Lucent USA respectively.

Fletcher [27] considers the case of the sinusoidally driven oscillator and shows (p. 29 and his Fig. 2.7) that
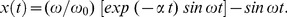
(3)For the case of the oscillator driven at its resonant frequency such that *ω* = *ω*_0_, the form of the response depends only on *ω*_0_ and *α*, or equivalently, *ω*_0_ and *ω*_0_/2*Q*. Eq. 3 is plotted in Fig. 2A for *ω*_0_ = 1 kHz and *Q* = 12.7, the quantities matching those derived experimentally by Shera and colleagues [29].

Inspection of Fig. 2A confirms that the driven oscillator takes *Q* cycles to build up to 0.96 amplitude when a stimulus at its natural frequency is applied (p. 26 of [27]; [32]), in the same way as the damped oscillator takes *Q* cycles to exponentially die away to *e^−^* ^π^≈0.04 when the sustaining energy is switched off. The number of cycles obviously depends on the amplitude criterion, and for the more usual half-power criterion (1/√2 or −3 dB), the build up time is *Q*/*π*, the result shown by Shera and colleagues [28] and indicated in Fig. 2A by the measure ‘b’. Reference [28] also shows how the half-power criterion is related to *Q*_10_ (the 10 dB criterion) and *Q*_ERB_ (the equivalent rectangular bandwidth), which are proportional to each other. Here, the half-power criterion is chosen, in which case the important result is that the driven oscillator takes *Q*/*π* cycles to respond to a stimulus at its natural frequency. A related consideration is the final steady-state amplitude reached by an oscillator when it is driven by frequencies off resonance. Again, this essentially depends only on the resonant frequency and *Q*. After normalising for the driving force and mass, the amplitude, *a*, is (p. 16 of [27]):
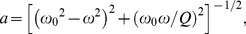
(4)and this function is plotted in Fig. 2B. A notable feature of this curve is the resemblance to actual measurements of the amplitude of basilar membrane vibration in response to a tone, and examples of this are shown in Fig. 2C and 2D for comparison. Figs. 2B–D show the distinctive asymmetry which reflects the behaviour of the harmonic oscillator when its displacement and driving frequency are plotted logarithmically. These plots show it is mistaken to say that the amplitude response of a simple resonator is symmetrical in the frequency domain (p. 154 of [33]).

High *Q* values mean that such resonators will take some time to reach maximum amplitude in response to a sound and they will also take time to decay afterwards. Therefore a classic argument against the resonance theory of hearing [34] is that such a cochlea will be unable to distinguish rapid changes in speech and music (it would be like a piano with the sustain pedal always on). It is not denied that there is a compromise between tuning sharpness and rate of stimulus discrimination, as Helmholtz himself was aware, but in practical terms the *Q* of the cochlea, about 30 at 10 kHz and 12 at 1 kHz, still allows very fast discrimination: a decay time of *Q*/π cycles translates to only about 1–3 ms.

### D. Phase delay and group delay of the driven oscillator

**Figure 3:**
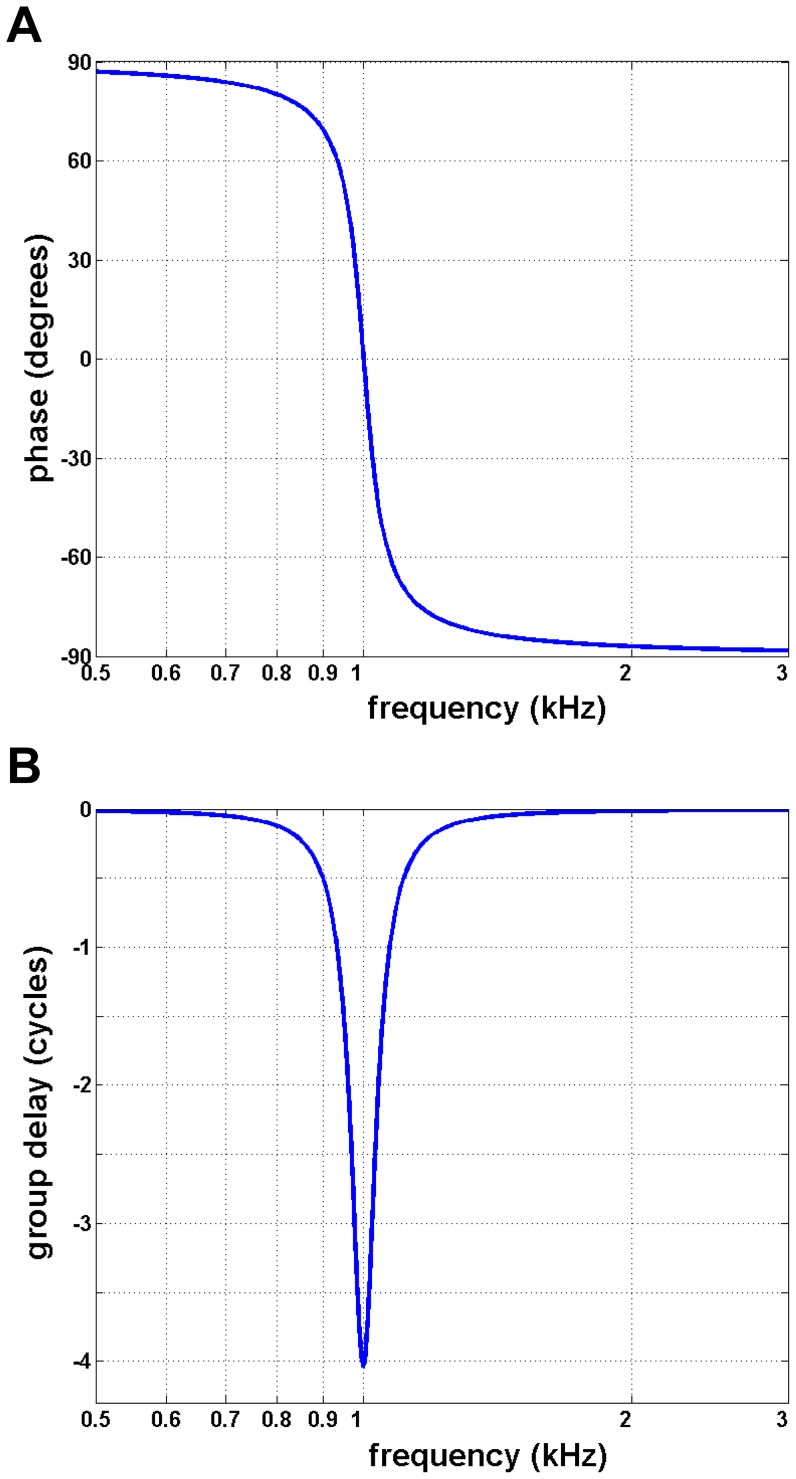
Phase delay and group delay of the driven harmonic oscillator. (**A**) Phase–frequency response of the same oscillator as in Fig. 2A, with phase given in terms of its velocity relative to the imposed force. At the resonance frequency of 1 kHz, the velocity is in phase with the force, whereas at the lowest frequencies, there is a phase lead of 90°; at the highest frequencies there is a phase lag of 90°. The total phase excursion is therefore limited to 180° (half a cycle). (**B**) Group delay of the same oscillator, which is defined as the slope of (A). Note that the maximum group delay of some 4 cycles (*Q*/π) occurs at the resonance frequency.

As well as the amplitude response, it is also informative to look at the phase response. Here it is important to distinguish two quantities, the steady-state phase delay and the dynamically relevant group delay. As shown by Fletcher (p. 16 of [27]), the phase of the driven oscillator, *θ*, relative to the driving force, is given by
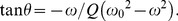
(5)Again, it is worth noting that the shape of the curve depends entirely on *ω*_0_ and *Q*. The phase delay for an oscillator of natural frequency 1 kHz and *Q* = 12.7 when subjected to a driving force of 1 kHz is shown in Fig. 3A. Note that below the resonant frequency the displacement leads the force by up to 90°, whereas above the resonance frequency the response lags the force by up to 90°. The total phase range is thus 180°, a well-known result.

Of particular interest for signal processing systems is the group delay, which gives the delay with which *information* can be delivered through a physical system [35]. The group delay, *T*_gr_, is related to the phase delay, *T*_p_, by [35], [36]:
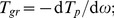
(6)that is, the group delay is the negative slope of the phase delay. Computing the derivative of Fig. 3A produces the curve shown in Fig. 3B. Here the group delay is shown in terms of the number of cycles of the driving frequency, and it is clear that the maximum group delay appears at the resonance frequency, where a delay of just over 4 cycles occurs. Note that this number is equivalent to the *Q*/*π* figure (time interval ‘b’) evident in Fig. 2A for the time taken for a driven oscillator to respond to a tone-burst at its resonance frequency; note also that this delay exceeds half a cycle, the limit for steady-state phase delay in a purely resonant system. This result will be returned to in Sections D and E-4 of the Discussion.

### E. Calculation of apparent wave velocity

Having calculated the response of a single driven oscillator, the next step is to see what the overall response of a graded oscillator bank looks like. When excited simultaneously, such as by an impulse composed of all frequencies, each element in the bank will take a different time to reach maximum amplitude, a time that depends on frequency and *Q*. The overall effect is to give rise to an envelope of activity travelling from where responses reach a maximum most quickly to where they are slowest. The analysis is restricted to the range 1 to 10 kHz, where data are readily available. The standard frequency–place map [37] gives the frequency and location of each resonator, and the *Q* values have already been specified (Eq. 2).

**Figure 4:**
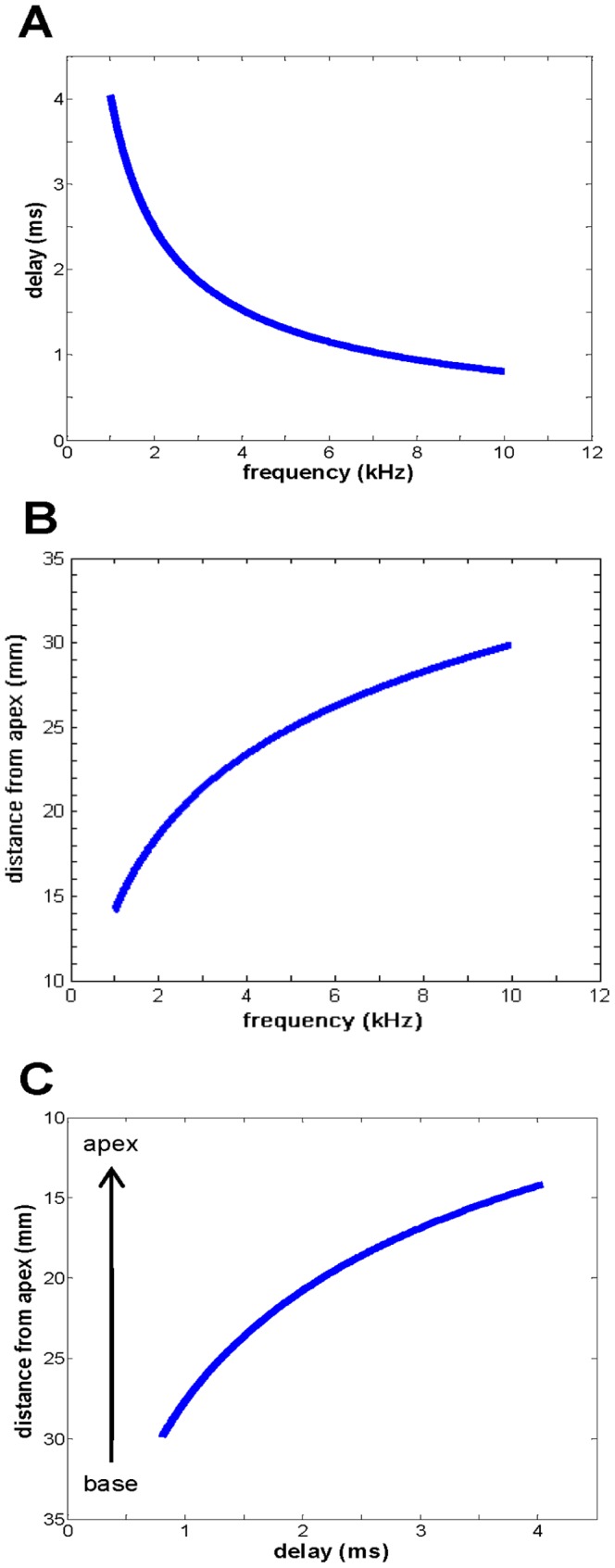
Delays in the cochlea in terms of frequency and distance along the basilar membrane. (**A**) Delay of cochlear resonators (ms) against characteristic frequency (kHz). (**B**) The Greenwood frequency–place map which relates distance from the apex to the characteristic frequency [37] over the range 1 to 10 kHz. (**C**) Cochlear delay as a function of distance. The *y*-axis is inverted so as to more easily appreciate that the wave is progressing (delay is increasing) from base to apex.

Based on Eq. (2), the delay at each point resulting from simultaneous excitation of all the cochlear oscillators can be simply calculated. Each oscillator will reach a maximum response after *Q*/*π* cycles; since one cycle occupies 1/*f* seconds, the time delay, *t*, at each characteristic frequency will be, in milliseconds,
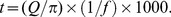
(7)This function is plotted in Fig. 4A, from which it is seen that the shortest delay (about 1 ms) occurs at the 10 kHz point and progressively increases towards the 1 kHz location, where a delay of 4 ms is found. This can be directly interpreted as a wave of activity appearing to move from base to apex, the same phenomenon as travelling wave theory describes.

To determine the speed of this apparent wave, the distances involved are needed, and this is a matter of referring to a map of characteristic frequency against distance from the apex. The well-studied frequency–place map [38] was first described by Greenwood (1961) [37], and has become standard in the field. The map, Fig. 4B, relates characteristic frequency in hertz, *f*, to distance in millimetres, *x*, from the apex. It is expressed as:
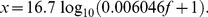
(8)


Together, these equations allow a plot of delay as a function of distance to be created (Fig. 4C). This depicts a wave progressing from base to apex, and the slope of the curve (*dx*/*dt*, which is velocity) indicates it is slowing down, a characteristic feature of the classical travelling wave [4]. Taking Equations 2, 7, and 8 together and differentiating shows that *dx*/*dt*, the velocity, *v*, of the travelling wave (in mm/sec) is given by (*dx*/*df*)·(*df*/*dt*), which as a function of *f* is:
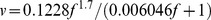
(9)or as a function of *x* by
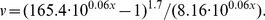
(10)


**Figure 5:**
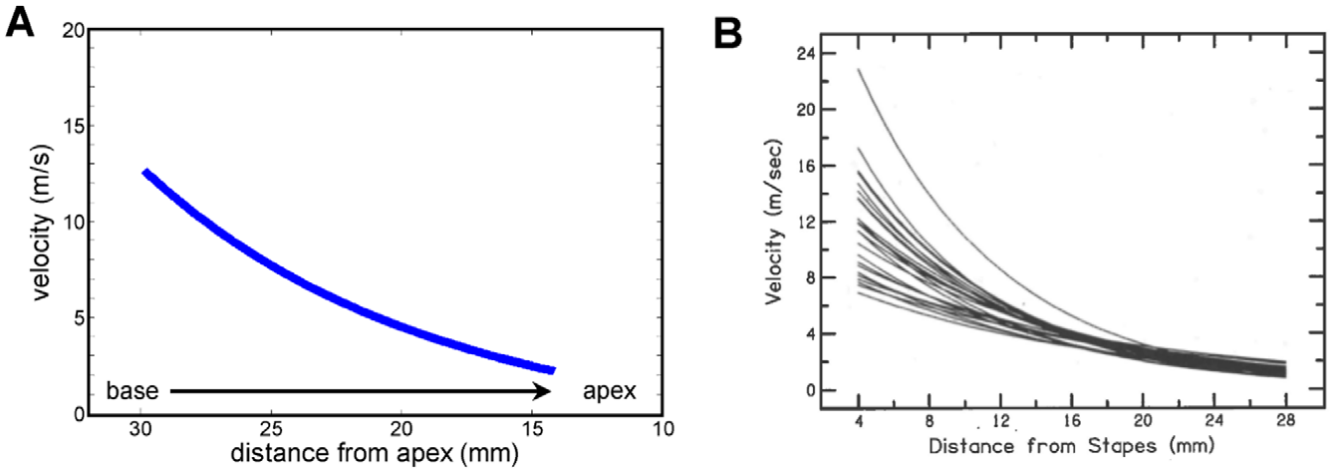
Calculated and measured travelling wave velocities. (**A**) Calculated apparent wave velocity along the cochlea in response to a simultaneous excitation of a bank of graded resonant elements. The wave starts at a speed of about 12 m/s at the basal (high frequency) end and slows to about 2 m/s at the apical (low frequency) end. These are typical travelling wave velocities. (**B**) Experimentally measured travelling wave velocity in 24 human subjects using ABR methods (from Donaldson and Ruth, 1993 [39], reprinted with permission of the Acoustical Society of America).

Equation 10 is plotted in Fig. 5A. It shows that the wave velocity begins at about 12 m/s at the 10 kHz point and slows down to about 2 m/s at the 1 kHz point. The significance of this curve is that it resembles actual travelling wave velocities determined experimentally. For example, Donaldson and Ruth [39] measured the latencies of auditory brainstem responses to different frequency bands on some 24 subjects, and their calculated travelling wave velocities are shown in Fig. 5B. The calculated velocities are remarkably close to experimental ones, even though values near the base are susceptible to wide variation. The derived velocities have not, of course, been adjusted for neural delays, nor has consideration been given to alternative threshold criteria in specifying build-up time. Nevertheless, this noteworthy result opens the way to re-interpreting cochlear mechanics purely as a resonance phenomenon.

## Discussion

The above calculations have shown that distinctive features of the cochlea – its frequency response, group delay, and apparent travelling wave velocity – which are usually explained in terms of a travelling wave model can be derived from basic resonance principles. Some major implications of these results will now be discussed, including a proposal for a new model of cochlear mechanics – a globally forced chain of phase-coupled oscillators – in which characteristic features of the resonance and travelling wave approaches are merged. The new model displays two distinctive aspects: its amplitude response is close to that expected from simple resonance, whereas its steady-state phase is generated from a combination of resonance and coupling along the chain. An important property of such a mechanical system, as documented in the literature, is that the amplitude and phase are largely independent. An appropriate electronic analog is presented as a basis for future modelling.

### A. Phase excursions of more than half a cycle

It has long been stated that, since the phase response of a simple resonator can be no more than ±90°, the resonance theory of hearing cannot be valid ([11], p. 199; [2], p. 461; [4], p. 145; [12], p. 16). While this is strictly true, it does not mean that a modified resonance model of some kind cannot be sustained. Moreover, on logical grounds, if it happens that a pure resonance model is inadequate then it still cannot be claimed that the travelling wave model (based on a transmission line) must necessarily be correct. There are alternatives, as the following text will explain. In a chain of phase-coupled oscillators, the phase delays in the system can be many cycles, but the individual oscillators can still be forced by a global stimulus that produces local resonance. It is suggested that in the cochlea there is competition for oscillators to synchronise with their neighbours and with the external forcing field, and it is this compromise which leads to phase delays exceeding 180°.

In seeing how this can come about, it is useful to clearly distinguish the dynamic group delay of a system and the steady-state phase delay at a point. Whereas the phase delay of a single resonator may have limits of half a cycle, its group delay can extend to *Q*/π cycles (as shown in Section D of the Analysis), and this has physical implications when a collection of individual oscillators are coupled into a single system. Physically, phase delay can be interpreted in terms of the delay of the carrier frequency, whereas the group delay is associated with that of the signal envelope and is the delay associated with the transport of energy and information through a system ([36]; Sect. 10.4 of [35]). Briefly: in signal transmission systems, the observable is the group delay and group velocity. Thus, in the case of light, the phase velocity can exceed 3×10^10^ cm/sec, whereas, of course, the actual signal must always travel at less than this speed and is measured as the group velocity. Similarly, in the cochlea, the response of the resonators is shaped by the basilar membrane on which they sit, forming an envelope whose relevant measure is the group delay. When a probe microphone measures the progressive phase delay of OAEs as frequency increases, or when a laser vibrometer measures the motion of the basilar membrane in response to a tone, these instruments use a constant sine wave to detect the phase delay, but it is the group delay – the negative slope of the phase–frequency curve – which most aptly specifies how the ear performs in terms of real-world sounds.

The idea being developed here is that the introduction of coupling to the individual resonators does not immediately destroy their resonance behaviour. What it does is join them into a unified system whose most relevant physical measure is their group delay. In a sense, coupling converts the group delay of the individual elements into the group delay of the whole. The individual elements are still able to respond to outside influences and to *resonate*, and the globally forced phase-coupled model put forward in Section E of the Discussion shows how this can be achieved.

When delays of otoacoustic emissions are measured (e.g. [40]), the most relevant measure is the group delay, and indeed the *Q* values used here [28] were derived from group delay measurements. This broad context has been confirmed by the experiments of Wilson (1992) [41] on sets of vibrating reeds, and it is this system which forms the basis for a model of the cochlea as a globally forced set of coupled oscillators. This approach is discussed in Sections D and E below. Wilson's model shows resonance, but it also exhibits extended cycles of delay. However, before describing this system, it is first helpful to define what is meant by a travelling wave system and show how this differs from a resonant one.

### B. Travelling wave and resonance

Given the calculations in the Analysis section which show that both travelling wave and resonance can in some major respects lead to similar outcomes, how is it possible to distinguish systems supporting travelling waves (such as ripples on a pond) from systems operating on the basis of pure resonance (such as the strings of a piano)? This section spells out the fundamental differences and demonstrates that the issue is more than one of semantics.

The core issue of travelling wave or resonance often becomes confused because travelling wave models are in fact built up of resonant elements [11], [12]. Crucially, however, in this situation the resonant elements are not isolated and driven in parallel, but driven sequentially by a slow wave on the basilar membrane. The analogy has been made to small masses floating on top of a water surface (Fig. 4.7C of [11]). In such an arrangement, each resonating element behaves like a small resonator sitting on top of a larger one (Fletcher, p. 218), and the driving force is slow and indirect, such as by a slowly propagating ripple on the surface of the water. The result is that, according to standard travelling wave theory, the serial wave progressively unloads its energy to the buoyant masses before reaching a peak and dying out before the resonant place is reached (p. 214 of [11]; footnote 10 of [42]; Fig. 3.7 of [12]). A clear difference between the travelling wave and resonance theories is therefore that the peak amplitude is reached *before* the resonance place in the former and *at* the resonance place in the latter.

Békésy made an attempt to try and clarify the mechanism powering the cochlear travelling wave, saying with his colleagues that “nothing is implied about the underlying causes” (whether a ripple along the membrane or a stimulus conveyed through the cochlear fluids) [43]; p. 16 of [12]. But the agnosticism didn't win many adherents because the heuristic simplicity of a hydrodynamically coupled travelling wave, in the face of no obvious resonating elements, remained appealing [14]. The discrete resonator idea of Gold [6], [8], [44] failed because at the time it was impossible to see how an active cochlea based on positive feedback to a local resonator – his regenerative receiver model – could work. From reading the recent literature, the impression may be gained that, semantically, the term “travelling wave” has been taken to mean any progressive motion without any concern about whether the primary stimulus is serial or parallel (e.g. in the debate between Dancer [45] and Ruggero [46]). This move has had the effect of losing precision in describing how the cochlea works because the unquestioned assumption is that there is a serial stimulus at work and that the appropriate model is the transmission line (section 3.4 of [12]; [47]).

**Figure 6:**
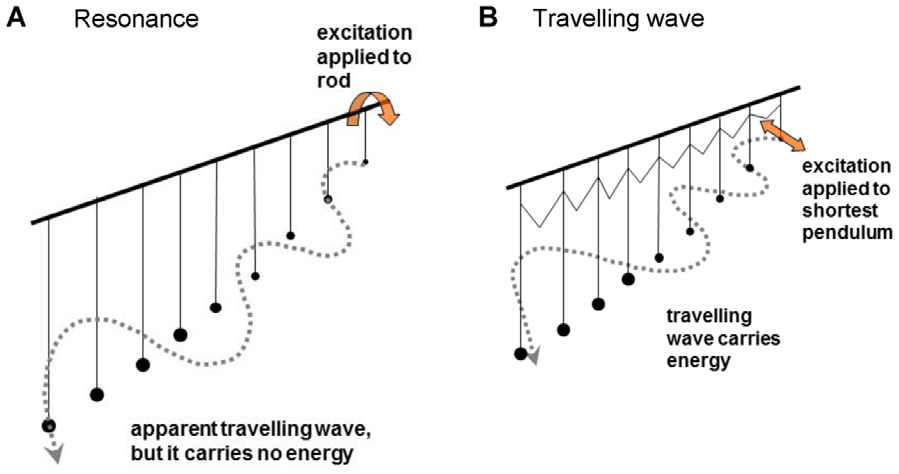
Békésy's pendulum analogy illustrating the difference between resonance and a travelling wave. In a resonant system (**A**), the pendulums hang from a common rod and are simultaneously excited by a short twist to the rod. In a travelling wave (**B**), the excitation is applied to the shortest pendulum and the energy moves progressively to neighbouring longer ones through rubber bands which supply coupling. In both cases a wave-like motion of the pendulums is seen.

To clarify the semantics and open the way to a fresh approach, it is helpful to look again at a nice distinction between travelling wave and resonance made by Békésy. He drew the analogy of a set of pendulums, of graded length, hanging on a rod [2](p. 519 ff). To create a travelling wave, the pendulums are connected by rubber bands, and the shortest pendulum is excited with a displacement (Fig. 6, right). Because of the coupling, the shortest pendulum excites each of its neighbours in turn, and a wave travels along the set, carrying energy. To illustrate resonance, Békésy pictured the same pendulums without coupling. To excite the pendulums simultaneously, the rod is given a sharp twist. Again, a wave propagates (Fig. 6, left), but in this case it carries no energy.

In terms of the cochlea, in the first case the stimulus could be a membrane-borne ripple, and in the second a fast fluid-borne compression wave stimulating all the cochlea's sensing elements nearly simultaneously. In one case the stimulus is *in series* with the bank of tuned elements and in the other it operates *in parallel*.

**Figure 7:**
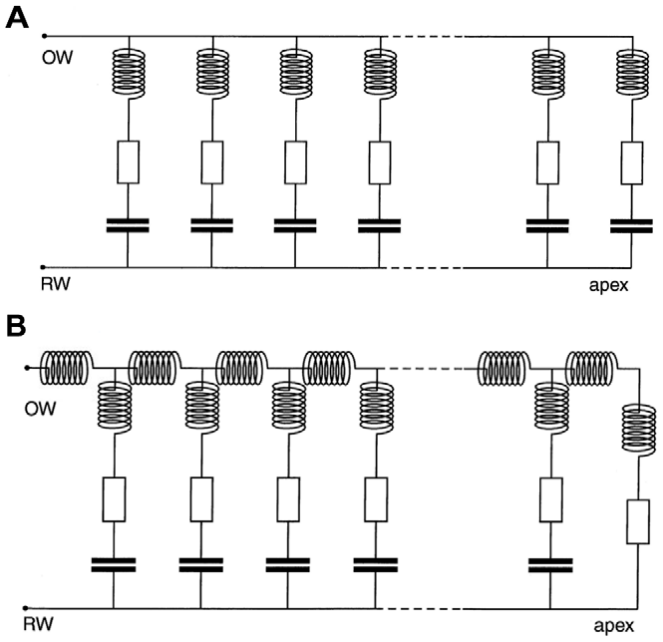
Comparison of electronic circuits representing resonance and travelling wave models. (**A**) A resonance model of the cochlea and (**B**) a transmission line or travelling wave model. Inductors are analogues of mass, resistors represent damping, and capacitors stiffness. In (A), each resonant filter element receives simultaneous input *in parallel*; in (B) each filter element receives a progressively delayed input signal as it propagates *in series* along the chain of coupling inductors. From p. 52 of Duifhuis [12] and used with permission of Springer Science+Business Media B.V.

The difference between travelling wave and resonance is graphically illustrated by Duifhuis (p. 52 of [12]) where the crucial distinction translates to whether or not there are coupling inductors between transmission line elements. In the resonance case (Fig. 7A), the input to the elements is in parallel; in the travelling wave case (Fig. 7B), the input reaches the individual elements in series through a chain of inductors, representing coupling by the mass of the fluid. In terms of general principles, the point has been made by Franck [48] that there are two distinct classes of oscillatory systems, force-dependent and flux-dependent, and the relationship between them is a matter of the direction of causality. In flux-dependent systems, the stimulus acts in series and the flux gives rise to forces; in force-dependent systems, the stimulus acts in parallel and the force produces fluxes. In Franck's terminology, therefore, the question is one of whether the cochlea is a force-dependent or flux-dependent system.

In the following section, the issue of parallel versus serial excitation is examined in the context of electronic models, and the two approaches are combined into a single circuit which displays aspects of both. This model might perhaps satisfy both sides of a long-standing debate.

### C. Electronic filterbanks

For many years a common way of describing cochlear mechanics has been in terms of electronic models, and the transmission line model has almost become the standard approach [9]. In these models, the cochlea's resonant elements are represented as a graded series of filters – a filter bank – and a good review of the advantages and drawbacks of various electronic models is given by Lopez-Poveda [49]. This author highlights a crucial distinction (p. 32): in the classical travelling wave interpretation of cochlear mechanics (e.g. [11]) the output of each filter serves as the input to the next, making the stimulus travel through the system *in series*. In contrast, some filter bank models (e.g. [50]) assume that all the filters share a common input signal, so that the elements operate *in parallel*. The distinction was also made by Duifhuis [51] who calls the first class “transmission line models” and the second “filter banks”. He points out that in the first class physical coupling is involved, whereas in the second the channels are independent. The two configurations, illustrated in Fig. 7, correspond to the two arrangements of pendulums in Fig. 6.

More recently, Lyon [52] has revisited the issue and speaks of cascade filter banks in the first case and parallel filter banks in the second. Unfortunately, the difference between the two – that they represent two very different underlying physical mechanisms – is not often emphasised because the general aim has been to achieve a phenomenologically satisfactory model, not a physiologically exact one. That is, the aim has been to get the filter shapes right to reproduce the psychophysical data without dwelling on the mechanics – in other words, to produce “the right output for a given input without paying much attention to the actual biophysical processes underlying a given physiological result” (p. 22 of [49]).

This blurring of distinctions has not helped to advance cochlear mechanics. The review of [49] includes a table in which all the surveyed models, transmission line and filter bank, are conflated by listing them as having a “filterbank (or equivalent)”. As it happens, parallel models now appear to be receding and the strong preference is for the transmission line model. In his recent paper, Lyon states that the parallel filterbank “would not have any natural relationship to traveling waves” (p. 3894 of [52]), and that the transmission line model is better, even if, in analogy with the flicked rope model of the travelling wave, it is necessary “to enforce conservation of energy” (that is, the stimulus energy must flow progressively along the membrane). The intent of the Analysis section was to show that such serial models are not always necessary and that it is equally possible – and even desirable if the physics so dictates – to describe cochlear mechanics using a resonance (parallel) approach.

In Section E below a way by which the dual aspects, serial and parallel, can be captured in a single model is set out, but as an introduction it first helps to describe a simple physical model that reflects such a duality of inputs and which seems to aptly represent the workings of the cochlea. The favoured analogy is a set of vibrating reeds driven by a magnetic field, and it was first described by Békésy and later investigated in some detail by Wilson [41]. In terms of the basic cochlear mechanics raised in this paper, it is a model having many virtues. It is a resonant system which also demonstrates travelling waves, and in major ways it comes closer to how this paper views the cochlea as operating – as a globally forced chain of coupled oscillators – than to the standard travelling wave model that Wilson thought he was describing.

### D. A system of vibrating reeds

**Figure 8:**
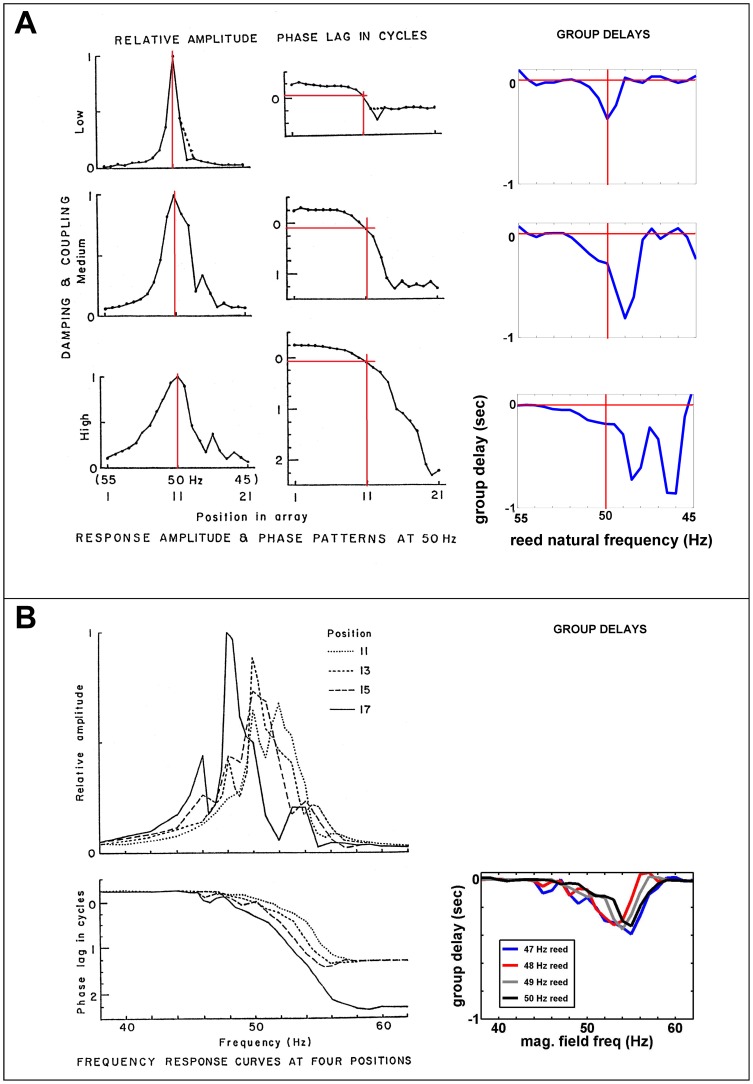
Properties of a graded bank of tuned reeds as an analog of the cochlea. The bank is a resonant system which, when coupled, also shows travelling waves. The reeds are driven in parallel by an oscillating magnetic field, and the plots in the left column are reproduced from Wilson (1992) [41] with permission from Elsevier and P. Wilson. (**A**) Relative amplitude and phase of the reeds in response to a 50 Hz magnetic field. At top is the purely resonant situation, without coupling; the relative width of the resonance peak (*f*/Δ*f*) provides a measure of *Q* (about 50). The phase delays show values expected from a driven oscillator, and the slope of the curve at the resonance frequency (red lines) gives the group delay, shown in the right column (about 0.4 sec, a value in keeping with *Q*/π cycles). In the two lower plots of (A), the reeds have been coupled together with a rubber band, first lightly (middle) then strongly (bottom), *creating a travelling wave in the band*. The *Q* values are now lower, about 35 and 25, and the group delays at resonance are also appropriately lower (0.3 and 0.2 sec), but resonance still occurs at 50 Hz where the amplitude is highest. Phase plateaus occur at delays of 1.25 and 2.25 cycles (exceeding the 0.25 cycles of the uncoupled case, but at frequencies far away from resonance, where amplitudes are low). (**B**) The lightly coupled situation again except that 4 individual reeds (natural frequencies 47–50 Hz) are driven at a range of frequencies to give amplitude and phase response curves. Again, the plots show that the reeds resonate (reach maximum amplitude) near their natural frequencies. Group delays are comparable to (A) and phase lags reach plateaus of 1.25 or 2.25 cycles. Although once more “travelling waves” occur in the rubber band, the primary event is resonance of the reeds.

An instructive demonstration of the similarities and differences between resonance and travelling wave has been given by Wilson [41] who studied the dynamics of a graded bank of tuned reeds – a Frahm frequency meter with 21 reeds tuned from 45 to 55 Hz. The Frahm reed system was also briefly investigated by Békésy [2]. A resonance situation was replicated by driving the free-standing reeds with a common 50 Hz magnetic field, and the travelling wave picture was modelled by using the same system supplemented with an intertwined rubber band which lightly coupled the reeds together. Although the experiment sought to highlight the differences, what becomes clear is that the behaviour in the two cases is actually closer than it first appears. Wilson's results are shown in Fig. 8. The top row of Fig. 8A illustrates the resonance situation and shows the steady-state phase of the free-standing reeds, *once oscillatory transients have died down some time after switch on*. It takes the expected form, with a range from +90° (at the 55 Hz reed) to −90° (at the 45 Hz one). The phase, revealed with a stroboscope, resembles the familiar response curve of a single driven oscillator, as shown in Fig. 3A (although note that the profile here is actually that of oscillators of different frequency driven by a fixed frequency).

To look at the effect of coupling, Wilson threaded a rubber band through his reeds in a similar way to how Békésy linked his set of pendulums. Light coupling is supplied by the stretch of the rubber, and the remaining text of this section aims to demonstrate that, in terms of a vibration sensor's detection abilities, the coupling makes only a minor difference.

After steady state is achieved in the coupled system, Wilson finds that the phase lag of the reeds now exceeds 1 cycle (Fig. 8A, middle row), and he draws attention to the fact that this is more than the half cycle seen before with the isolated resonators. The appearance of this extra shift is meant to illustrate the formation of a travelling wave and how the system is now behaving quite differently to before. Certainly, it proves that the system is now more complex than second-order, but the thing to note is that in major respects the system is little changed. There is now an envelope formed by the rubber band, and a wave appears to be continuously travelling along it from the high-frequency end to the low-frequency end, but in terms of each of the individual underlying resonators, their sympathetic resonance in response to the driving magnetic field is much the same. One difference is that after transients have died out the rubber band carries ‘a travelling wave’, whereas without it the reeds' vibration envelope is static; however, in both cases the reeds within both envelopes are still vibrating, especially those near the 50 Hz point.

A feature missing from this picture is the behaviour of the reeds between the time that the electromagnet is switched on and the time that quasi-static conditions are achieved. Importantly, in both situations (coupled and uncoupled), each reed, driven by the 50 Hz magnetic field, goes through a pattern of increasing amplitude, similar to the response of the driven oscillator shown in Fig. 2A.

In this situation, *the group delay* is a useful descriptive measure. The group delay has been defined in Eq. 6, and at the resonance frequency this is little affected by light coupling, as computation of the phase slope, −ΔΦ/Δ*f*, from Wilson's data (right column) demonstrates. Wilson also looks specifically at 4 reeds (of natural frequencies 47, 48, 49, and 50 Hz) and drives them with magnetic fields of 38 to 62 Hz, measuring the amplitude and phase at each frequency (Fig. 8B). As Section D of the Analysis showed, the slope of all the phase curves is a direct measure of the group delay at frequency *f*, so that at the resonance frequency, about 50 Hz, all systems – coupled and uncoupled – show a group delay of 200–400 ms or 10–20 cycles. In other words, after the electromagnet is switched on, the resonators begin building up amplitude and it takes about one-third of a second for them all to reach a quasi-static condition. Since group delay in cycles equates to *Q*/*π*, this means that the associated *Q* was about 30 to 60, values that roughly match the width at half-power of the amplitude curve (*f*/Δ*f*) in Fig. 8A.

Importantly, in the one-third of second before steady state is achieved, apparent waves of excitation – travelling waves of a sort – formed by envelopes of displacement will also progress along the bank of resonators (see, for example, the remarkable pendulum waves seen in a Harvard University Natural Sciences Lecture Demonstration [53] at http://www.youtube.com/watch?v=yVkdfJ9PkRQ). In fact, the time between switch-on and steady state is the crucial period to examine, for this describes the time course of how the reeds – proxies for the sensory function of the hair cells – are excited. The question most relevant to the cochlea is how the reeds build up amplitude in response to the driving magnetic field, *not* their behaviour after steady state conditions have been achieved, and this is where Wilson's experiment can be said to miss the point. Perhaps an apt analogy is the pendulum video played backwards, so that all the pendulums start at rest and then gradually build up amplitude, driven by an invisible force. If this were done, we would see a slow build up and many travelling wave envelopes moving along the set of pendulums, governed by the phase difference between them (see also the discussion of pseudowaves in Section E below).

The rubber band has forced all the reeds to conform to a travelling wave envelope, which is visible, whereas without it the reeds follow the dictates of the electromagnet alone and there may or may not be a definable “envelope”. Near resonance, however, where amplitudes are large, the underlying reeds behave very similarly whether they are coupled or not. In both cases, they are vibrating with large amplitudes near their natural frequencies, and the peak amplitudes are reached after about *Q*/*π* cycles. Most importantly, the reeds continue to be driven by the magnetic field; *it is not true* that the reeds, considered as analogs of sensory hair cells, are “deflected” or supplied with energy as a travelling wave moves along. Said another way, the vibrating reeds create the travelling wave; the travelling wave does not vibrate the reeds (albeit that after steady state has been achieved the wave does affect vibration of the off-resonance reeds, but these have small amplitude). The dominant energy supply continues to come from the oscillating field – it is the primary stimulus in the causal chain – and the rubber band is secondary, simply defining the displacement envelope. Moreover, the group delays and *Q* values at resonance are not greatly changed by the rubber band, as examination of Fig. 8 shows.

It is possible to be impressed with the large change in phase between the reeds at either end of the array, which increases as the coupling becomes stronger. *After switching-on transients have dissipated*, the phase difference between the low frequency reeds (near 45 Hz) and the high frequency reeds (near 55 Hz) grows from half a cycle (the uncoupled condition) to 1¼ or 2¼ cycles. As frequently pointed out, such a phase change exceeds what is possible with a single resonator. However, it is less frequently noted that *before steady state is reached* there can easily be more than 1 cycle of delay in a resonant system comprised of a graded bank of multiple, independent resonators. For example, as shown in Fig. 3, with a *Q* of 13 at the apex, there is up to *Q*/π (∼4) cycles of group delay, whereas at the base, with a *Q* of 30, there is up ∼10 cycles of delay, giving potentially many cycles of “waves” travelling from one end to the other (again, the video demonstration is helpful in appreciating this). At the same time, it is again also worth noting that the end-most reeds, which exhibit the largest phase changes, carry small vibration amplitudes (they are off-resonance), so that most of the system energy continues to reside near the middle, strongly resonating reeds. In this reed model, it is simply not true that the travelling wave has “died out” before reaching its resonant place (the 50 Hz reed), as a classical low-*Q* transmission line model would predict [11], [33], [54]. Although the rubber band serves to define the moving envelope, by itself the energy it carries is small.

In summary, the coupled and the uncoupled reeds behave quite similarly. At the resonance frequency, the coupled and the uncoupled reeds are still very much vibrating at high amplitude, driven by the electromagnet, and the beguiling appearance of a wave in the rubber band *after* steady state has been achieved should not blind us to the essential similarity of the coupled and uncoupled cases, especially *before* steady state is achieved. From the results shown in Section E of the Analysis, it should be plain that the apparent travelling wave velocity in a purely resonant system is very much the same as that in a coupled system provided the coupling is light – the *Q* remains high – and the resonant frequencies of the underlying resonators are not appreciably changed. Only the off-peak behaviour is noticeably different. Interestingly, the phase curves (middle column of Fig. 8A) are remarkably smooth below, at, and above the resonance frequency, showing no sign that forcing by the 50 Hz oscillating magnetic field has created any discontinuity in the reeds' phase response at this frequency, although the amplitude of course has been much increased; this distinctive feature will be picked up again in the next two sections.

**Figure 9:**
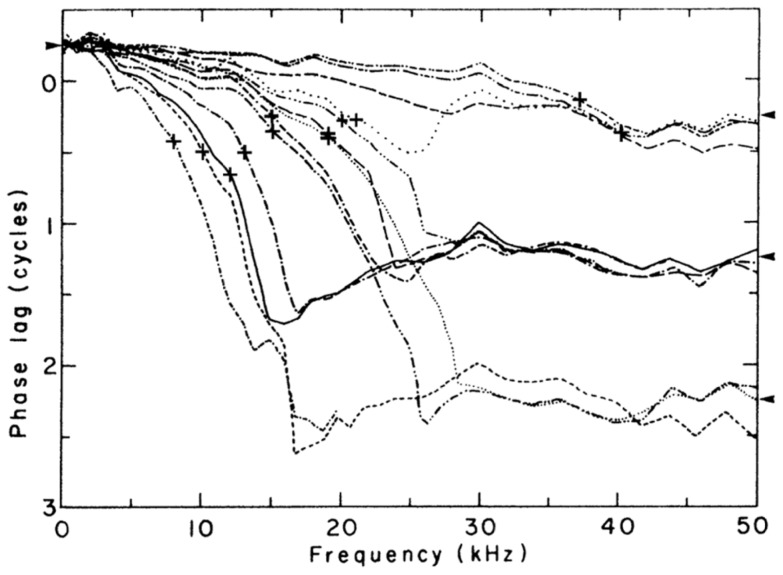
Representative phase–frequency plots for the cochlea of the live cat. The plots show the response of a single point on the cochlea as measured by Wilson and Evans [136] using a capacitive probe. Each characteristic frequency (CF) is marked with a cross (+). The right-hand arrowheads mark integer number of cycles of phase lag from −90°. Note the similiarity of these phase–frequency plots to that of the Frahm reed (Fig. 8) in which there is forced resonance of the reeds. This similarity supports the view that the CFs are places at which cochlear resonance is occurring. A difference, however, is that in the cochlear case the phase lags at CF (crosses) appear at values greater than 0° (but still less than 1 cycle). It is suggested that this phase lag is due to the dynamics of forced coupled oscillators: each point must compromise between synchronising with the external force and with its different-frequency neighbours (p. 126, p. 276 of [62]). Reproduced with permission of the authors.

The end-point of this discussion is that a set of Frahm reeds, or any bank of graded resonators, can, at a basic level, be well described in terms of its resonance properties, of which group delay is more important than steady-state phase delay. A similar conclusion was reached in a recent paper by Babbs [55] which considered the resonance behaviour of the basilar membrane represented as an uncoupled set of masses on springs. Quantitative analysis of this (high *Q*) Helmholtz-like model showed an apparent travelling wave moving from base to apex in response to a click, as well as in the first 4 ms after the onset of a 1 kHz tone. However, Babbs was unable to physically reconcile this travelling wave, and its many cycles of delay, with the results obtained using continuous tones, which showed only the expected steady-state solution of one half-cycle of delay (his Fig. 9, which show the results of homogenous solutions and particular solutions to the wave equations). The difference between Babbs's particular and homogenous solutions is the counterpart of the difference between the steady-state and transient solutions of the driven oscillator (see section 2.8 of [27]). Once this difference is recognised it becomes unnecessary to look, as Babbs did, for the lingering effects of small repetitive impulses to explain observed large delays and travelling waves in cochlear mechanics. It is now possible to understand Békésy's finding that the difference between the resonance and travelling wave theories “disappears completely” for transients (p. 542 of [2]). Appreciating the crucial role that group delay plays in the excitation of a driven oscillator helps in understanding this “surprising” result and in seeing what this may mean for individual outer hair cells and for otoacoustic emissions in general. To understand the transient response of any system, the group delay is the key parameter and the appearance of a travelling wave, transitory or continuous, is not important. In the case of the cochlea, the persistent travelling wave that appears in the steady state is only of secondary interest. Some typical cochlear response curves are shown in Fig. 9, and the resemblance of certain key features to Fig. 8 will be discussed in the next section.

This paper takes the view that the vibrating reed frequency meter is an excellent analogue of how the cochlea works, closer in fact than Wilson himself portrayed and better than has been acknowledged. The set of vibrating reeds is a ‘flux-driven’ system operated by the magnetic flux permeating the reeds. An important insight is that *it is not the case* that the travelling wave in the coupled rubber band causes vibration of the reeds. The travelling wave is just an interesting side effect of a magnetic field driving a parallel filter bank.

**Figure 10:**
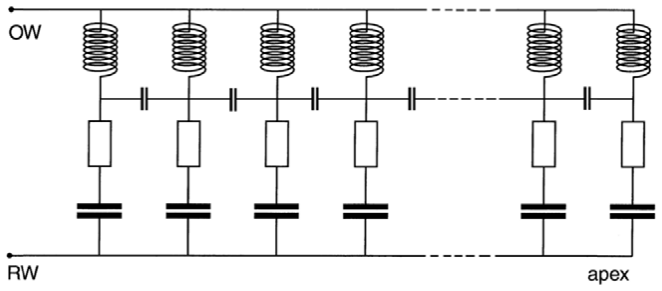
A hybrid circuit representing the cochlea as a globally forced set of coupled oscillators. It is a combination of the two circuits in Fig. 7. Each element is simultaneously forced by the signal rail, while stiffness coupling between sections is represented by small capacitor linkages. The model can also be viewed mechanically as a vibrating reed frequency meter or as a set of coupled pendulums which are simultaneously excited via motion of the suspension rod, merging key aspects of Fig. 6A and 6B.

To conclude this section, the difference between the travelling wave and resonance models was illustrated in Fig. 7 in terms of equivalent electronic circuits [12]. In the resonance case, the input to each tuned element appears in parallel (Fig. 7A); in the travelling wave case, the input reaches the individual elements in series through a chain of inductors (Fig. 7B). After having observed the behaviour of the vibrating reed frequency meter, and seen that it does not function like a transmission line but more like a bank of resonators, it is suggested that an appropriate electronic model of the cochlea is a hybrid of Figs. 7A and 7B, which is set out in Fig. 10. Like the Frahm frequency meter, it combines parallel inputs to the resonant elements, which makes the input to each element continuous and instantaneous, while at the same time providing coupling between adjacent elements. More explicitly, in terms of Békésy's pendulum models it means there is a third option which the literature has not specifically addressed: the pendulums are coupled but *their excitation comes from motion of the suspension rod*. Anticipating discussion in the next section, the hybrid model embodies a key property – forcing – which is seen as essential in making the cochlea behave resonantly, while including neighbour-to-neighbour coupling that reflects the physical disposition of the resonant elements on the cochlear partition. The following section will show that the hybrid model can be analysed in terms of a chain of coupled oscillators undergoing global forcing.

### E. Chains of coupled oscillators

Wilson's insights into cochlear mechanics were gained using a physical model, a set of reeds vibrating within a magnetic field. Describing this physical analogue in mathematical terms would be invaluable because then the behaviour of the system could be systematically explored. Babbs [55] has analysed the situation for a graded bank of resonators without coupling, but the coupled case is more complex. Coupling undoubtedly gives rise to ‘a travelling wave’, but a difficulty is to define the governing factors and identify the parameters involved in its propagation. As set out below, there are different types of travelling wave, some relying on point-by-point transmission of energy, such as a ripple on a pond, but at the other extreme are ‘pseudowaves’ that carry no energy whatsoever. Where does the cochlear travelling wave fit in?

The most common approach to modelling travelling waves has been to look to the case of the electronic transmission line (Fig. 7B), but this limits the full range of possibilities because it emphasises serial transmission and downplays parallel phenomena, the very factors this paper wishes to emphasise. For the set of vibrating reeds, the key property to be included in the modelling is the *forcing* action of the oscillating magnetic field, and in the case of the cochlea it is the parallel forcing exerted by acoustic pressure on all the OHC resonators, and this aspect is the focus of this section. The standard transmission line model fails to accommodate parallel forcing; moreover, it also enforces ‘conservation of charge’ [17] so that basilar membrane motion is compelled to conserve fluid volumes as it deflects vertically. These conditions need not apply in the cochlea, particularly if the sensing cells are pressure sensitive and the resonating fluid parcels oscillate radially (across the partition) [26], not vertically as the standard model presupposes (Ch. 3 of [12]).

The following examines the general case of a chain of coupled oscillators with a linear gradient in natural frequency and with external forcing. Travelling waves are produced, but the point to be emphasised is that they can just as easily be associated with the apparent waves we examined in the Analysis as with the waves produced by the transmission line model.

Coupled oscillators are an important topic in physics, chemistry, electronics, and biology, and they have been the subject of an immense amount of work [56]–[62]. The general mathematical problem of oscillator-generated wave phenomena is set out by Murray [61], where the sequence of *N* coupled oscillators is described by his equation 12.19 (p. 428) as *N* equations of the form:
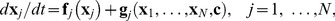
(11)where **x** *_j_* is the amplitude of the *j*-th oscillator, **g** *_j_* represents the coupling effect of the other oscillators (in the case of global ‘all to all’ coupling, there will be *N*−1 oscillators; if only nearest-neighbour coupling, there will be 2), and **c** is a vector of coupling parameters. These equations are intractable without some simplifying assumptions, and it turns out that, with weak coupling, the *amplitude* of the oscillators is not important, only their *phase* (see numbered subsection 1 below). In this situation and without forcing, the equation above can be reduced to [56]:
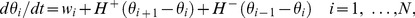
(12)where *θ_i_* is the phase of the *i*-th oscillator and *ω_i_* is its natural frequency. *H* ^+^ and *H* ^−^ are functions representing the combined effects of oscillators *i*+1 and *i*−1 on the *i*-th oscillator, and they can be computed numerically.

With a forcing term added, such as to represent the action of an oscillating magnetic field on a set of Frahm reeds, the equations can be expressed [63], [64] as
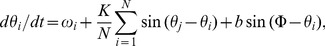
(13)where Φ is the phase of the forcing, *d*Φ/*dt* its frequency, and *b* its strength.

Many efforts have been put into analysing chains of forced and unforced oscillators, and they are complex systems, exhibiting rich dynamics [56], [60], [61]. One aid to understanding is that, in general, a given set of oscillators can be considered to behave like a single oscillator interacting via its mean field (p. 98 of [65]). An inference is that a group of oscillators mutually entrained at a given frequency will have an effect on the other non-entrained oscillators as if there were external forcing at that frequency.

Although interactions in chains of coupled oscillators can involve either ‘all-to-all’ coupling or nearest-neighbour coupling, in the cochlea there are mechanisms that might permit both types to occur. However, for a chain graded in frequency, it is simpler and seems more apt to focus on the second possibility, which Eq. 13 represents when *N* = 3. Such coupling supports the proposed analogy with the vibrating reed frequency meter. Some of the differences between the two arrangements are spelt out in ref. [64].

Various approaches have been made to solving Eq. 13; see for example Fig. 1.14 in Section 1.2.5 of [66], where the frequency response curves for varying degrees of forcing are shown, the thesis of Rhoads [67], and similar work [68]–[71]. Despite the complexity of the system (which can lead to chaos under sufficiently large forcing), intuition suggests that in a graded bank of oscillators, forcing will give rise to resonance in those oscillators whose natural frequencies come close to that of the driving frequency (see the ‘natural’ approach of Harvey [69]).

A large amount of the literature on coupled oscillators has focused on the analysis of phenomena seen within systems of oscillating chemical reactions [60], and this literature can be given immediate relevance to the cochlea by noting that, in the governing equations, chemical diffusion is a direct counterpart to elasticity in a physical system (p. 141 of [60]). This means that diffusion in the well-studied Belousov–Zhabotinsky reaction is analogous to the rubber band in the set of Frahm reeds, and the results can be carried across, including travelling wave fronts. From the results of an extensive search of the literature it would seem that the dynamics of the coupled vibrating reed system has not been specifically analysed. However, after consulting the general literature on coupled and forced oscillators, four key features of such systems stand out as particularly relevant to the cochlea.

#### 1. The dominant role of phase differences

In modelling coupled oscillators an outstanding factor is that the system is almost totally driven by phase differences between adjacent oscillators. Winfree's wide-ranging book [60] is a full examination of what patterns can emerge as a result. The dominant effect of phase is also the basis of Kuramoto's description of coupled oscillators (Ch. 3 of [59]). If the phase difference between an oscillator and its two neighbours is paramount, then it is generally the case that the faster oscillator always drives the slower one (p. 381 of [57]). Taking the case of Wilson's vibrating reeds, then it can be seen that there is a progressive phase shift from the faster reed to its slower neighbour, so there will be a cumulative phase lag from one end to the other when the reeds are coupled, and this is exactly what Wilson describes. Pikovsky and colleagues explain (p. 126 of [62]) how each oscillating point has to compromise between synchronising with the external force and with its differing-frequency neighbours.

In Kopell's analysis (Eq. 12), it is notable that the amplitude of the oscillators does not enter into the equation. Phase differences alone drive the system, and when phase accumulates sufficiently it leads to phase plateaus at multiples of half a cycle. Wilson's data shows such a plateau for the Frahm reeds (Fig. 8) and, of particular interest, he draws attention to the similar phase plateaus that occur in the cochlea (Fig. 9). The plateaus occur at 1 or 2 complete cycles below the equivalent purely resonant system, and this explanation of the plateau in terms of the phase differences between coupled oscillators requires closer investigation (see also the solutions found by Manevich and Manevitch, e.g., p. 23, where stationary points recur at intervals of −π/2±2kπ). At the same time, it should be noted that a standard explanation for phase plateaus observed in the cochlea has not been agreed upon [72], [73].

Because the amplitude does not affect the phase, and vice versa, both these quantities can be treated as independent quantities. Another way of expressing this is that for a weakly coupled system, amplitude is stable whereas phase is free (p. 32 of [62]). External forcing can be applied to one end of the chain or the other, or the middle, and it will not appreciably affect the behaviour of the rest of the system because coupling between neighbouring elements is the most important factor (p. 181 of [56]). Similarly, external forcing of all the oscillators (such as by a magnetic field) should only affect the amplitude near the resonance frequency of the field, and this is apparent in Wilson's results. In Fig. 8A, it is clear that the phase of the travelling wave does not deviate as it passes through the resonance frequency at 50 Hz (middle column), even though the amplitude at resonance (left plot) is more than an order of magnitude larger than off-resonance.

#### 2. Appearance of travelling waves

The general result from solving Eq. 13 is that *if the oscillators are graded in frequency, then a travelling wave of activity will always progress from the oscillator with the highest frequency to that with the lowest* ([59], [66]; p. 179 of [56]), just as Wilson observed in his system. See also p. 388 of [57], Sect. 13B of [60], [61], [74], [75]; Fig. 9.14 of [71]. This occurs regardless of the strength of the coupling parameter, although the governing equations are easier to solve with weak coupling [58].

There has been a range of work, beginning with [76], in which the cochlea has been modelled as a chain of coupled oscillators. There are related studies in which spontaneous otoacoustic emissions (SOAEs) have been successfully modelled as the dynamics of a forced chain of van der Pol oscillators [77]–[79]. However, most of these treatments have incorporated standard transmission line (travelling wave) assumptions into a basic local oscillator (resonance) model. However, indications from the present work are that transmission line properties and similar assumptions (like conservation of volume from basilar membrane displacement) are not essential in order to generate what appear to be travelling waves. A full analysis of the inherent possibilities and limitations of both travelling wave (serial) and resonance (parallel) stimulation is needed. In particular, the emergence of wave fronts and other dynamic travelling-wave like phenomena from a simple phase-coupled chain is one aspect that calls for attention.

#### 3. Appearance of phase plateaus

The occurrence of distinctive phase plateaus has already been touched upon, but it is worth documenting the general finding that, depending on the strength of coupling, phase (and frequency) plateaus are common when chains of coupled oscillators are modelled (p. 205 of [58]; [78], [80]–[82]). Wilson's reeds exhibit just this phenomenon (Fig. 8), as does the cochlea (Fig. 9 and [73]), and it appears to have a more natural explanation in terms of general oscillator dynamics than in terms of the more restricted transmission line model. Again, it is worth noting that the phase plateaus have generated a degree of controversy and there is no agreed explanation [73].

#### 4. Forced entrainment vs mutual entrainment

A distinctive feature of chains of coupled oscillators subject to external forcing is that there is competition between forced entrainment, due to the driving field, and mutual entrainment due to coupling between the oscillators [63], [83]–[85]; p. 126 of [62]. As an outcome of this competition there will be a phase difference between the two sets of oscillators, with each set trying to pull the other into its orbit [86], [87]. In effect, the two sets of oscillators behave like two oscillators of differing frequency which, when coupled together, each try to entrain the other [88], [89]. If the coupling is sufficiently strong, the oscillators will become tightly synchronised, but if the coupling is weak, there will be a compromise [85], [90]. Because of coupling, the compromise frequency will deviate from the individual natural frequencies [63], [91] and, importantly, there will be a phase lag between the entrained oscillators and the external driving force [82], [85], [86], [89].

The major point is that in a coupled Frahm reed-like system, the global magnetic field will only be more or less successful in entraining a particular reed – because the reed has neighbouring reeds also wanting to entrain it. The result is that there will be a phase difference between the resonant reed and the external magnetic field (which will, as evident in Eq. 13, depend on the strength of the field). Indeed, there is a phase lag of about 45° in the 50 Hz reed shown in Fig. 8. However, more generally, the literature shows that phase lags occur between an entraining force and a resonant oscillator in a chain because the frequency of the oscillator has been modified through its coupling to neighbouring oscillators [79], [85]. Applied to the cochlea, the suggestion is made that the extra steady-state phase delay observed in situations like Fig. 9 is due to the coupling effects of nearby oscillators. Instead of the resonant frequency (the CF) occurring near zero degrees to the applied tone (as it would with pure resonance), it occurs in Fig. 9 at a phase lag of roughly half a cycle, and, more generally, the literature shows phase lags at CF of 0.5 to 2 cycles [73].

Another interesting feature of Fig. 9 is that the characteristic frequencies (the crosses which mark peak response amplitude) appear on sections of the phase curve that are relatively smooth. At the same time, the plateaus tend to have phase lags that are half-integer (or integer) number of cycles larger than those at CF. In these respects, the system resembles the Frahm reed system of Fig. 8 and also, more generally, chains of coupled oscillators driven by external forces. The compromise discussed earlier could explain certain deviations in phase from the strict integer values; moreover, as expected from a model of forced oscillators, the actual measured phase delays at CF do depend systematically on sound intensity (Fig. 8 of [73]). The suggestion being made is that in the cochlear case the non-zero phase lag at CF might be the result of a coupling compromise, and that the measured responses could in fact reflect resonant-like forcing of a graded and coupled bank of oscillators. One constraint on the phase curves is worth noting: following Section D of the Analysis, the negative slope gives the group delay and so at a resonance frequency *f* this must equate to *Q*/π cycles (or *Q*/*f* π seconds). The slope at CF thus reflects the mechanical *Q* of the underlying resonance, and slopes of the curves in Fig. 9 point to realistic cochlear *Q* values for the live cat (e.g., the curve with CF of 13 kHz has a group delay at CF of 0.3 ms which corresponds to 4.8 cycles and therefore a *Q* of about 15).

The most apt treatment of synchronisation of oscillators by external tones is by Vilfan and Duke [79] who show how the frequency of an oscillator when subject to an external tone can be altered by mutual coupling with its neighbours. Vilfan and Duke modelled a set of coupled oscillators under a frequency gradient, reflecting the arrangement in the lizard ear. They used numerical techniques on a set of active Ginzburg–Landau oscillators to investigate how the animal's spontaneous otoacoustic emissions might arise via coupled oscillators and to examine the effect of external tones. Their modelling showed how an external tone could either raise or lower the frequency of an oscillator (compared to its natural frequency) depending on the nature of the coupling with its neighbours: elastic coupling between the oscillators raised the frequency, whereas dissipative (resistive) coupling could either raise or lower the frequency (Figs. 2–4 of [79]). When irregularities and noise were added to simulate more realistic conditions, the external tone dragged the frequencies of nearby oscillators either up or down compared to their natural frequencies (Fig. 5 of [79]), but a general finding was that maximum phase locking occurred at a frequency below that of the external tone (Fig. 7 of [79]).

A factor to again keep in mind when trying to explain the extra half cycle of delay in Fig. 9 (compared to the resonant situation) is the possible disjunct between the motion of basilar membrane displacement, which experimenters measure in the vertical direction, and the motion of an oscillating fluid parcel which might occur in the radial direction (as a result, perhaps, of active outer hair cells [26]). As well, there could be appreciable phase delays between the motion of outer and inner hair cells, and one model of this process calculated that the delays here could amount to several cycles (Fig. 12 of [92]).

Once more in the context of the lizard ear, a later paper by Gelfand and colleagues [93] also provides insight into the way a coupled set of oscillators can mimic cochlear function. The authors modeled the gecko's cochlea as a coupled chain of 110 van der Pol oscillators and found that the response of the system, when the elements were coupled, could be made to match observations of frequency clustering in the animal's spontaneous otoacoustic emissions. Notably, the authors examined the phase of a viscously coupled system against time (their Figs. 2A) and found that “the most striking phenomenon” was “the presence of waves of synchronization that advance in both directions along the array” (p. 5 of [93]). They were at pains to declare that the waves “in no way imply the presence of traveling waves on the basilar membrane” (*ibid*.), which in one sense is true, but in another, the waves are travelling entities which actually could be recorded by appropriate equipment. They repeated the simulation with elastic coupling, and “waves of synchronized oscillation” again appeared (their Fig. 3A), but this time only flowing from the high frequency end to the low frequency end. A distinctive (but unremarked) feature is that the speed of the wave (as determined by the slope of the arrow in their Fig. 3A) was some 1.3 m/s, a value not unlike the ABR-derived travelling wave velocities seen here in Fig. 5B, and underlines the importance of defining what is meant by a travelling wave and its causal origins.

The unifying point is that the plots shown in Figures 2A and 3A of [93] show just one aspect of the oscillators' behaviour – *phase* – and that if the corresponding *amplitude* was plotted as a function of time, the build-up in response to a stimulus might indeed replicate the cochlear travelling wave – that is, it could show an amplitude peak occurring some *Q*/π cycles after stimulus onset and that the envelope of this peak might appear to move from base to apex at just the velocity calculated in Section E of the Analysis.

The paper by Gelfand and colleagues goes on to simulate the effect of an external tone by using a forced van der Pol oscillator model. The authors find that the oscillators under such global forcing synchronise to the imposed tones (their Fig. 6), and this frequency entrainment is the same behaviour as displayed by the vibrating reed cochlear model described in Section D immediately above. Despite structural differences, the remarkable operational similarity of the lizard cochlea and the mammalian cochlea is emphasised in [94], and the wide-range of literature outlined in the present section demonstrates that the mechanics of the mammalian ear can be well simulated without needing to take a transmission line approach. Gelfand and colleagues also compared their results to the earlier Vilfan and Duke paper [79] and were somewhat surprised (p. 10 of [93]) to find that parameters such as mass, viscosity, and elasticity did not appear to make major differences to the calculated outcome. This might be understood by appreciating the general result, summarised in the text above and modelled in [79], that the dynamics of chains of coupled oscillators are primarily driven by phase differences in the system, so that the key controlling factor is the frequency gradient.

Despite the “compelling” resonant-like behaviour in the lizard ear, Vilfan and Duke [79] were reluctant to draw parallels with the mammalian cochlea, which they considered considerably more complex “as a result of the propagating wave on the basilar membrane”. A recent modelling paper by Wit and van Dijk [78] simulates the mammalian situation, and here the authors examine whether human SOAEs could be produced by a chain of coupled oscillators and what the effect of forcing with an external tone would be. Their paper is generally congruent with the resonant forcing mechanism put forward here, and their work does explain some aspects of SOAEs, even though the authors again consider their model to be too simple to explain cochlear function. Nevertheless, their work does demonstrate that forcing at frequencies of 1505 Hz and 1513 Hz (their Fig. 7a) acts in competition with a mutual entrainment frequency of 1509 Hz. If attention were directed to the phase shifts associated with this forcing, there is the potential to explain major features of the human cochlea's phase response, which resemble those seen in Fig. 9. Parallels with Wilson's vibrating reed system (Fig. 8) could be explored, and it might be possible to place the hearing of lizards and mammals in a similar framework [94].

#### 5. Pseudowaves, phase waves, kinetic waves

A most interesting finding to come from the coupled oscillator literature is the description of how a wavefront appears to move along a phase gradient, a phenomenon related to what has been described in the Analysis for the moving amplitude peak of cochlear resonances. This has been called a ‘pseudowave’ by Winfree [60] and Murray [61], a ‘phase wave’ by Kuramoto [58], and a ‘kinematic wave’ by others [95]. In all cases it is an apparent wave produced by the coordinated phase cycling of a bank of oscillators or ‘clocks’ – Winfree makes an analogy to the “ball of fire” arising from a chain of flashing strobe lights at the end of a runway (p. 237 of [60]) – and unlike conventional travelling waves it carries no energy.

Winfree first documented the properties of pseudowaves in a series of papers analysing the Belousov–Zhabotinsky reaction, where the wave shows up as moving bands of blue and red that propagate through a solution that is undergoing a cyclic chemical reaction (see summary in [60]). Starting with a chain of synchronised clocks, he showed that the speed of a pseudowave is simply the inverse of the phase gradient, −1/(*dφ*/*dz*)τ, where τ is the period, *φ* is the phase, and *z* is the distance. For example, if the phase gradient is 0.01 s/mm, then the wave will appear to travel at 100 mm/s. Obviously, the speed of this ‘travelling wave’ has no limit – it can even be faster than light if the phase gradient is shallow enough – and it will pass straight through any physical barrier erected in its path because nothing is actually being transported. There will be no reflection, and if two pseudowaves move in opposite directions and collide, they annihilate each other. In a petrie dish of reactants, the wave becomes two-dimensional and spirals emerge, but a similar analysis applies [96].

By considering the diffusion of reactants, a degree of coupling is introduced, and as mentioned before, chemical coupling is mathematically the same as elasticity in a physical system (p. 252 of [60]). The combination of pseudowave and diffusion gives rise to something called a trigger wave by Winfree [97], who advocates that they be carefully distinguished (even though there is a steady transition from one to the other [98]). Of relevance to cochlear mechanics, the rate of reaction is governed by temperature, so that a system which is hotter at one end than the other is analogous to a bank of graded pendulums. The temperature gradient will therefore produce a phase gradient, and a pseudowave will result. Note also that, unlike a physical wave, this pseudowave cannot undergo reflection.

Murray [61] also draws attention to the implications for biology of chains of coupled oscillators, and one of his models is the row of pendulums all hanging from the same horizontal rod but with a gradient in their periods. If they are simultaneously stimulated, once more it will look as if a wave is travelling along the pendulums – a pseudowave – with a wavelength decreasing with time. He draws together chemical reactions, central pattern generators, and nerve impulse propagation with a similar set of equations, emphasising the importance of phase differences in each system.

Taken together, this discussion of coupled oscillator chains underlines that there is more than one way of generating a travelling wave, and when one is observed in the cochlea, it does not necessarily mean that the transmission line model is the only possible explanation. Such a wave may indicate the presence of an energy-carrying ripple analogous to a pulse in a transmission line, but it could also be a moving phase front driven by phase differences in a resonantly forced system, just like the set of vibrating reeds in a Frahm frequency meter. The conclusion is that the forced vibrating-reed system presents a much closer analogue to the cochlea than has generally been appreciated, and it calls for further investigation.

### F. Coupling in the cochlea

All travelling wave models invoke coupling, through either the fluid or the membrane, and such coupling cannot be entirely avoided. Nevertheless, the demonstration by Wilson (Section D of the Discussion) shows that the practical difference between coupled and uncoupled cases can be slight, especially at the resonant place (the characteristic frequency) and before steady state is reached. Yet the difference has been used to dismiss the insights that the resonance picture offers and to argue that adherence to a travelling wave formalism is necessary [6], [11], [12], [33], [99].

Békésy examined what happened to thin membranes when they were subjected to vibrating needles (pp. 540–541 of [2]), and this illustrates the functional effects that coupling can have. The response of a thin membrane depends on whether the longitudinal coupling is weak, giving *Q* values over 10, or strong, with *Q* values nearer to 1. The first represents *Q* values in an active cochlea and the latter a passive or dead one. Further insight into the effect of coupling is provided by a paper [100] that uses a novel approach to cochlear modelling in which hydrodynamic coupling can be explicitly represented by a single parameter, *c*. When *c* = 0, there is no coupling and no travelling wave, only resonance. As coupling increases, a travelling wave emerges. Notably, the travelling wave velocity is shown to depend directly on the coupling, and the wave slows down as coupling increases. Another approach is to treat the fluid coupling and the basilar membrane dynamics separately [101], and then bring them together algebraically. This approach can give conventional travelling wave behaviour, but of particular interest this passive model can also produce pure resonance (Fig. 12c of [101]) when the basal half of the basilar membrane is prevented from moving. This result can be understood in terms of the travelling wave being the *envelope* of the responses of individual resonant elements and that neighbouring envelopes tend to blur what is going on underneath – the activity of the individual resonators – unless the neighbouring responses are somehow suppressed.

Coupling does lead to a travelling wave envelope that will carry some energy, but the claim being made is that this energy, as in the Frahm reed case, will be relatively small; in the case of the cochlea the amount carried by the envelope – the basilar membrane – is likely to be insufficient (in the normal high-*Q* situation) to be a major cause of hair cell stimulation. The causal chain is in fact the other way round: first comes the hair cell stimulation that leads to resonance, and then this secondarily creates an apparent travelling wave.

Observations of large delays do not exclude some sort of resonance behaviour; it could be that the phase of the individual resonators (possibly a triplet of outer hair cells) has escaped detection and that only the peak of the basilar membrane motion has been sensed after several cycles of resonator build up. This idea is not new, and was put forward, for example, by a collection of papers in 1989 in which the ITER (International Team for Ear Research) presented results endeavouring to show that the response of the basilar membrane was considerably less than that of the outer hair cells themselves [102]; see also [103]. They concluded (p. 12) that “vibrations of the basilar membrane [and the bony shelf] may both be produced as a consequence of hair cell vibration.” Indeed, as long ago as 1937, the suggestion was made that the outer hair cells were stimulated directly by sound, not by movement of the basilar membrane [104], although Békésy was later of the opinion that “direct stimulation of the sensory cells by compressional waves is unlikely” (p. 128 of [2]).

Although an uncoupled set of resonators can give rise to what appears to be a ‘travelling wave’, it is worth reiterating that the underlying physics in the two situations differs, and therefore the underlying stimulus chain and associated mathematics will also be different. The discussions between Nobili et al. [13] and Shera et al. [14] are illuminating in showing how one physical model can have multiple mathematical formulations. From that discussion it seems that the mathematics describing a resonant system driven by a fast pressure wave more closely approaches what is set out in ref. [13] than in ref. [14]. Consider that any pressure sensor in the cochlea – say one based on compressibility of the outer hair cells – will invariably act bidirectionally and therefore induce pressure changes as well as detect them (in this context it is worth noting that some have interpreted OAEs as the instantaneous sum of outer hair cell activity [105]). Thus, at some level, it may be necessary to consider, as Nobili and colleagues do, all the “myriad individual oscillators, each interacting with the others instantaneously through the fluids” [14](p. 354), and that there is “no wave propagation delay between the BM oscillation at any given place and its contribution to the force detected by the stapes” [105](p. 348). Looked at another way, the motion of Békésy's uncoupled pendulums will indeed act back on the supporting rod and thereby affect all the other pendulums. Further investigation of such all-to-all coupling appears warranted.

Another dimension to the discussion is the question of what is the exact causal mechanism by which the fast pressure wave interacts with the hair cells at their characteristic frequency and causes resonance. It could be that OHCs intercept pressure waves through possessing some compressible intracellular material (see [94], [106]). Alternatively, it may still be possible to retain the conventional picture of stereocilia deflection as the initial stimulus, and keep a picture of a locally resonant basilar membrane, by supposing that the pressure difference across the membrane, set up by the fast wave, somehow bends stereocilia directly (without requiring the usual travelling wave envelope). A problem with this alternative is that to retain a locally resonant basilar membrane, the coupling would need to be spatially very small, and this may be difficult to achieve. Which process is the effective stimulus remains for experiment to determine, although this paper inclines to the compressibility mechanism.

At this point, a historical discussion of resonance theories of hearing, and detailed arguments for and against them, could be undertaken, but instead the reader is referred to Chapter 2 of ref. [18], where a comprehensive account is given. Standing out from that survey is the work of Dancer [45], who appears to have come close to the ideas set out here. He concludes (p. 310) that “the cochlear partition appears to behave in the same way as a bank of resonators of which all the elements are excited simultaneously by the acoustic pressure”. Dancer's ideas were challenged by Ruggero [46], but only by blurring the distinction between travelling wave and resonance and largely returning the argument to a semantic one. The question will not be resolved until the underlying terms are clearly distinguished, and a major aim of the present paper is to move discussion in this direction.

### G. Zero signal-front delay in cochlear mechanics

A good way of distinguishing whether travelling wave or resonance operates in the cochlea is to examine three different types of delay: signal-front delay, group delay, and total delay. The properties of group delays have already been noted (see Analysis Section D and Discussion Section D). However, signal-front delay is unique to travelling wave models and deserves close attention. Signal-front delay corresponds to the travel time of a signal [36], and in the case of the cochlea this is the time for the stimulus to propagate from the stapes to the individual hair cell [40], [107], [108]. For a resonant cochlea driven by a fast pressure wave, the travel time is effectively zero, so the only significant delay in cochlear mechanics is the resonance build-up time or filter delay (which produces all the group delay). This issue has been systematically addressed by various workers [40], [107]–[112] and the relationships between the delays are labelled by the letters *a*, *b*, and *c* in Fig. 2A.

In a travelling wave system, the total delay *c* is the sum of the signal-front or propagation delay *a* and the group delay *b*. The measure *b* is also called the filter delay, envelope delay, or resonance build-up time [111]. In a resonant system, however, the fast pressure wave sweeps through the 30-mm-long cochlea at a speed of 1500 m/s, making the signal-front delay, *a*, only microseconds and for practical purposes zero. Of course, allowance for transduction delay needs to be made when cochlear nerve recordings are done, and this is usually reckoned to be only about 1 ms [113].

An important finding from Siegel and colleagues [111] is that, after considering the wider literature and allowing for neural delay, the signal-front delay as measured by SFOAEs was less than 0.1 ms for frequencies higher than 2 kHz and somewhat more for lower frequencies (their Fig. 2). The signal-front delay was 0.5 ms (0.5 of a cycle) at 1 kHz, but rose to more than 1.5 ms at 330 Hz (still less than 0.5 cycle). The intent of Siegel and colleagues was to challenge the theory of coherent reflection filtering (by comparing measured delays with twice the basilar membrane delays), but given the potential errors in estimating synaptic delays, a stronger statement can be made: that the results are very close to what is expected from a pure resonance model.

The conclusion that signal-front delay is practically zero is strengthened by other findings in the literature, and these will now be addressed.

An important study is that of Whitehead and colleagues [114] who measured the onset latencies (to the −3dB peak) of distortion product OAEs in the time domain and compared them to phase-gradient latencies (group delays). A crucial finding was that, in general, both measures were about the same, meaning that the signal-front delay must have been almost zero. As the authors express it, “a substantial portion of the rise time attributable to the ear reflects the effects of filtering within the cochlea” (p. 1675). Indeed, with an observed delay of 4–5 cycles attributable to the cochlear filters, this translates to an equivalent *Q* of 12–15, a reasonable number.

Whitehead and colleagues also note that the DPOAE delays they observed were quite similar to those for TEOAEs and SFOAEs and that these can be generally matched to cochlear travel times based on electrophysiology, again implying that practically all cochlear delays are filter delays.

A complementary study is that of Konrad-Martin and Keefe [115], who again used time–frequency methods to look at the build up and decay of SFOAEs in response to tone-bursts. They defined the onset latency as the time for an OAE to build up to its −3 dB point, typically 6 ms, but their time–level plots reveal how arbitrary this definition is. The levels are plotted logarithmically, and on this scale the level rises almost linearly from the noise floor. Of particular interest, when the levels are extrapolated backwards (their Figs. 4f, 5f, 6f, 7f), they intercept the noise floor *at about 0 ms*. This is just what we expect to see from the driven harmonic oscillator: a ring-up of the amplitude over *Q*/π cycles. Indeed, the authors were puzzled by this rising trend, as it was contrary to “the assumption that the onsets of the direct generated OAE source [and the secondary reflection] … would each be abrupt” (p. 2039). On the other hand, the work gives direct support to a resonance interpretation and weighs against travelling wave models. All OAE delays appear to be explainable in terms of group delays of resonators having specific *Q*.

Temchin and colleagues [113] found that signal-front delay, measured on the basilar membrane, was only 0.2 cycle at 100 Hz (the apex), rising to 0.5 cycle at 1 kHz, and dwindling again at higher frequencies. At 10 kHz the delay was just 18 µs (0.2 cycle). Similarly, Narayan and coworkers [116] considered that DPOAE group delays reflect only the filter delay at the basilar membrane, with signal front delays of only 25 µs. Ren and colleagues measured group delays of DPOAEs [112] and found that values were nearly equal to those measured at the stapes. de Boer and Nuttall [117] concluded that travel time in a real cochlea is small compared to resonance build-up time.

A later paper by de Boer [118] reinforces the point, noting that responses from the base of the guinea pig cochlea can be well represented by a minimum phase filter and that travelling wave delays are unnecessary. In earlier work by this author [119], [120], revcor functions were fitted to cochlear nerve data and it was found that the delays, after accounting for synaptic delays, were measurable in microseconds. As an elaboration, the 1989 paper also measured the order of the equivalent filters and found that the order required was at least 2 (that of a simple mass–spring system) but sometimes higher (4 or even 10 at high frequencies), meaning that at the level of the nerve a simple resonator is an oversimplification and a higher-order filter may be necessary.

In summary, the indications are that, in terms of basilar membrane mechanics, a basic resonance model can go a long way in explaining the cochlea's workings. Travel time can generally be neglected or accommodated within the error associated with measuring group delay.

### H. The active cochlea

The finding of cochlear echoes [5], [6] created a revolution in our understanding of the inner ear, providing unmistakeable proof that the organ is an active device, not a passive one, and forcing auditory science to reconsider all previous cochlear theory [6]. Kemp and later researchers have endeavoured to construct an active cochlea model on top of a passive travelling wave model, and have assumed that the observed otoacoustic delays embody the time for a travelling wave to travel to its characteristic place, a filter build-up time, and the time for a “reverse travelling wave” to return to the stapes [121]. The standard theory of the active cochlea, that of coherent reflection filtering (due to Zweig and Shera [19], [20]), assumes that the delay between stapes and characteristic place can be recycled multiple times, although filter build-up time remains a factor. Most models find that the amplification provided by outer hair cells needs to be restricted to a small region near the peak [122], which is puzzling in the travelling wave picture but a natural outcome of a resonance model.

Emphasising the importance of filter build-up time, and of particular relevance to the results raised here, it is significant that Shera and colleagues [28] find that the *Q* of the cochlea can be expressed in at least 4 species as
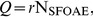
(14)where N_SFOAE_ is the response delay in stimulus frequency emission cycles and the factor *r* is a so-called ‘tuning ratio’. The ratio is approximately 1 for humans above 1 kHz (and in other species above 3 or 4 kHz). Even in a lizard, a similar relationship was found [123], although in this case the tuning ratio was around 1.6 over most of the range tested. The reason for divergences at low frequencies is outside the scope of this paper. The main point is that a direct connection between *Q* and the number of cycles of build up and decay, as set out in Eq. 7, is just what one expects to see in a graded bank of uncoupled harmonic oscillators. The work here highlights this connection and shows how far a simple resonance model can go in accounting for basic aspects of cochlear mechanics: frequency response, time delay, and travelling wave velocity. The elusiveness of reverse travelling waves also becomes easier to appreciate [94].

### I. Anatomical origin of Q

A feature of the calculations of travelling wave velocities is that they are all based on using specific *Q* values. Once the *Q* values have been set, the rest of cochlear mechanics naturally follows. This suggests there is something special about the way that cochlear *Q* originates. A detailed focus on this point will not be made here, but it is worth noting that a resonance model of the cochlea has been presented [124] in which the *Q* values derive from the geometry of the organ of Corti. In this surface acoustic wave (SAW) model, wave fronts reverberate between the rows of active outer hair cells and the *Q* derives anatomically from the tilt of the OHC lattice in the plane of the reticular lamina. The tilt means that, geometrically, each cell will sustain two resonating cavities of different lengths – and hence different frequencies. In turn, the difference in frequencies naturally specifies the *Q*, and so each hair cell will carry its own fixed sharpness of tuning.

### J. Hearing without the travelling wave

The work here has been directed towards constructing a resonance model of the cochlea in which the travelling wave is seen as more of an appearance, an epiphenomenon, than as a causal agent. It might therefore be useful to compare mammalian ears and their travelling wave with the ears of reptiles, which are generally acknowledged to lack such a mechanism [123], [125], [126]. Reptiles have their sensing cells sitting upon a stiff support and so a travelling wave – in the sense of a propagating motion that bends stereocilia – is thought not to operate in these creatures. On the other hand, the characteristics of reptile ears are “strikingly reminiscent” of those in mammals [123], and so it is possible that both classes of animal use the same mechanism – resonant detection of a fast pressure wave stimulus – in order to hear. Rather than extend the text here to investigate this possibility, the reader is referred to a recent paper [94] where this aspect is specifically addressed; this reference also sets out a model of how the detection mechanism may operate similarly in both classes of animal. If reptiles and mammals indeed use the same basic mechanism to hear, this would support a resonance approach to mammalian hearing.

## Conclusion

A graded bank of uncoupled resonating elements, with appropriate *Q* values, has been found to have properties similar to those that were previously seen to be signatures of travelling waves. The frequency response and the group delay near the characteristic frequency are similar to observations, as is the apparent travelling wave speed. Together with literature reports that the front delay is practically zero, these properties suggest that, in an active cochlea, it is not necessary to have a serially coupled travelling wave as the causal stimulus, and that a fast, parallel-acting stimulus could be equally effective.

The hypothesis put forward here is that the active cochlea might operate as a graded bank of resonant elements driven by a fast pressure wave in which case a travelling wave could arise simply as a secondary effect (an epiphenomenon akin to the pseudowave of Winfree [60]). In such a model there is no serial excitation, no coupling between the elements, and no energy carried by the wave. On this view the vibrating reed frequency meter, in which all the reeds are driven in parallel by an oscillating magnetic field, forms a very good analogy to the way the cochlea functions, perhaps more apt than has so far been appreciated. The vibrating reed model was first raised by Békésy, but only Wilson seems to have studied it since in any detail. Although relatively simple physically, its behaviour reflects that of a graded set of phase-coupled oscillators under global forcing, which can be represented as an electronic circuit (Fig. 10).

A notable property of such a system is that the oscillators' phase and amplitude are largely independent. This means that the individual oscillators are still able to be resonantly excited by external forcing, giving rise to appreciable group delays. The resonance occurs close to the forcing frequency, but there is some extra phase delay at resonance due to a degree of coupling and a compromise between an oscillator's pull towards mutual entrainment and forcing entrainment.

Although a broad sketch of the mathematics involved in analysing a set of globally forced phase-coupled oscillators has been given, a thorough analysis in the context of electronic circuits and vibrating reed frequency meters – and their translation to the mammalian cochlea – still needs to be done. Winfree has explored the astonishing complexity of coupled oscillators in his book [60], and this work in particular opens the door to a full alternative treatment of cochlear mechanics. The extensive prior work on wave propagation in excitable media becomes accessible once it is recognised that elasticity in a mechanical system plays a role analogous to diffusion in a chemical one ([60], p. 141).

If a resonance-based approach to the cochlea is adopted, and the traditional forward travelling wave is actually closer to Winfree's pseudowave – whose motion relies largely on a negative phase gradient – it follows that a backward travelling wave in the cochlea is not possible [94]. Instead, otoacoustic emissions can be considered as being carried by fast pressure waves from the cochlea's bank of graded resonators (which have a positive gradient of group delay from base to apex).

This wide-ranging investigation has highlighted the ambiguity behind the term ‘travelling wave’. In the past, the term has been used to describe any arrangement where there is a moving wave front. This confounds two situations. The first is where the wave-front carries energy and operates as a serial stimulus; this is the familiar usage in physics and is the one emphasised in cochlear mechanics to date. The second meaning is to describe a wavefront (specified by either its phase or its peak amplitude) that carries no energy and has no causal power in itself – it arises from a parallel stimulus to a resonant system and can be likened to a pseudowave (or its coupled counterpart, the trigger wave).

In the context of the cochlea, this paper favours the second interpretation. The evidence assembled shows that resonance need not be a wildly incorrect description of cochlear mechanics, although it has often been portrayed that way. Just because a phase delay of more than 180° is observed does not automatically mean that a resonance mechanism (and its parallel stimulus) must be totally discarded, and it by no means implies that a serially activated, energy-carrying model – along the lines of a transmission line – is necessarily correct. The present work favours the interpretation that a tone produces global, near-simultaneous forcing in a graded bank of coupled resonators and this causes an apparent travelling wave. However, such a wave carries virtually no energy and is not the main causal stimulus for hair cells.

A major shortcoming of the standard travelling wave theory is that it fails to take forcing into account – that an oscillating force could act in parallel on all the cochlea's resonant elements. Instead, travelling wave theory promotes the idea that the stimulus energy is progressively dissipated as it propagates sequentially along the basilar membrane. The standard model tends to generate the possibly mistaken belief that there is a serial causal chain, leading to ideas like there being a power flux from base to apex (e.g., [127], [128]; Section 3.6.1 of [12]) or that electronic analogues based on serial filter banks are appropriate (e.g. [47]).

Possibly, then, Gold and similar travelling wave skeptics were on the right track, and outer hair cells can be simultaneously stimulated by the fast pressure wave – they do not have to wait for a travelling wave to reach them. The picture could be that resonance happens at the hair cell level and that this activity is then coupled to the basilar membrane underneath. Otoacoustic emissions can then be interpreted as fast pressure waves that give an instantaneous signature of the aggregate activity of all the resonators.

The motivating force of this synthesis has been the power and elegance of Helmholtz's resonance model (p. 404 of [2]) and to highlight some of the conceptual difficulties associated with the conventional travelling wave picture. Of course, both models have value in reflecting certain aspects of cochlear function, and ultimately there needs to be a “reconciliation of the theories of the two giants of auditory physiology, H. von Helmholtz and G. von Békésy” [129]. Such a synthesis might be done along the lines of Fig. 10 where there is a “signal rail” that allows for parallel forcing of all the resonant elements by the fast pressure wave. It is possible that the hair cells might detect the fast wave by some deformation that in turn leads to bending of stereocilia, but another possibility, favoured here, is that the body of the outer hair cell is pressure sensitive, allowing stimulation to occur directly [94], [106], [130], [131].

The clear advantage of the resonance picture is that it is simple, and a reverse travelling wave is not needed to describe what is going on. Simple second-order resonators – harmonic oscillators – do have limitations [119], [120] and at some stage it will be necessary to consider higher order systems with dispersion and nonlinearities, particularly for DPOAEs [12], [40]. However, it is possible to show that every nonlinear model can be given a linear equivalent [132], and it is in many ways remarkable that parallel-based models can give results fairly similar to serial-based equivalents. Nevertheless, in terms of choosing one model or another, it will be experiment that decides between them.

In comparing travelling wave and resonance, it is useful to take note of an argument Gold put to Békésy: any attempt to use a “peak detecting” mechanism to refine a broadly tuned system always faces a serious problem in discriminating signal from noise [133]. Gold used this principle to argue against the broadly tuned travelling wave which underpinned Békésy's model. Békésy contended that the sharp tuning observed in the cochlear nerve was the result of some neural peak-detection mechanism [34]. The same logic can be used with some force to argue that any active transmission line model of the cochlea using cascaded amplification stages will strike the same problem and will become swamped by noise. Indeed, models of the cochlea using cascaded arrays of electronic circuits (e.g. the 120-section electronic analogue of [47]) have encountered just this difficulty: very high gain factors and problems with noise.

In summary, the question, once thought settled, has now become one of determining exactly what is the effective stimulus to the outer hair cells – is it a slow propagating ripple set up by trans-membrane pressure, or is it a fast fluid-borne pressure wave? The question left unanswered by Wever, Lawrence, and Békésy – whether the travelling wave is an entity arising from stimulus energy passing rapidly through the cochlear fluids or slowly along the basilar membrane – has again resurfaced.

The distinction between resonance and travelling wave has often been blurred, and over the years it has given rise to unwarranted conclusions. On the other hand, a way forward may be found in viewing the cochlea as a graded set of globally forced coupled oscillators. This model provides common ground between the two schools of thought, and gives a clear place for resonance as well as for basilar membrane coupling. Reconciling these two historically disparate approaches could bring us to a better understanding of how the cochlea works.
